# Genetic correlation between chronic sinusitis and autoimmune diseases

**DOI:** 10.3389/falgy.2024.1387774

**Published:** 2024-09-24

**Authors:** Enze Wang, Yingxuan Sun, He Zhao, Meng Wang, Zhiwei Cao

**Affiliations:** ^1^ Department of Otolaryngology Head and Neck Surgery, Shengjing Hospital of China Medical University, Shenyang, China; ^2^ Department of Neurology, The First Affiliation Hospital of China Medical University, Shenyang, China

**Keywords:** Mendelian randomization analysis, chronic sinusitis, genome-wide association study, rhinitis, allergic, asthma

## Abstract

**Objective:**

The association between autoimmune diseases and chronic rhinosinusitis in observational studies remains unclear. This study aimed to explore the genetic correlation between chronic rhinosinusitis and autoimmune diseases.

**Methods:**

We employed Mendelian randomization (MR) analysis and linkage disequilibrium score regression (LDSC) to investigate causal relationships and genetic correlations between autoimmune phenotypes and chronic rhinosinusitis. Additionally, transcriptome-wide association (TWAS) analysis was conducted to identify the shared genes between the two conditions to demonstrate their relationship. The CRS GWAS (genome-wide association study) data and other autoimmune diseases were retrieved from ieuOpenGWAS (https://gwas.mrcieu.ac.uk/), the FinnGen alliance (https://r8.finngen.fi/), the UK Biobank (https://www.ukbiobank.ac.uk/), and the EBI database (https://www.ebi.ac.uk/).

**Results:**

Utilizing a bivariate two-sample Mendelian randomization approach, our findings suggest a significant association of chronic rhinosinusitis with various autoimmune diseases, including allergic rhinitis (*p* = 9.55E-10, Odds Ratio [OR] = 2,711.019, 95% confidence interval [CI] = 261.83391–28,069.8), asthma (*p* = 1.81E-23, OR = 33.99643, 95%CI = 17.52439–65.95137), rheumatoid arthritis (*p* = 9.55E-10, OR = 1.115526, 95%CI = 1.0799484–1.1522758), hypothyroidism (*p* = 2.08828E-2, OR = 4.849254, 95%CI = 1.7154455–13.707962), and type 1 diabetes (*p* = 2.08828E-2, OR = 01.04849, 95%CI = 1.0162932–1.0817062). LDSC analysis revealed a genetic correlation between the positive autoimmune phenotypes mentioned above and chronic rhinosinusitis: AR (rg = 0.344724754, *p* = 3.94E-8), asthma (rg = 0.43703672, *p* = 1.86E-10), rheumatoid arthritis (rg = 0.27834931, *p* = 3.5376E-2), and hypothyroidism (rg = −0.213201473, *p* = 3.83093E-4). Utilizing the Transcriptome-Wide Association Studies (TWAS) approach, we identified several genes commonly associated with both chronic rhinosinusitis and autoimmune diseases. Genes such as TSLP/WDR36 (Chromosome 5, top SNP: rs1837253), ORMDL3 (Chromosome 13, top SNP: rs11557467), and IL1RL1/IL18R1 (Chromosome 2, top SNP: rs12905) exhibited a higher degree of consistency in their shared involvement across atopic dermatitis (AT), allergic rhinitis (AR), and chronic rhinosinusitis (CRS).

**Conclusion:**

Current evidence suggests a genetic correlation between chronic rhinosinusitis and autoimmune diseases like allergic rhinitis, asthma, rheumatoid arthritis, hypothyroidism, and type 1 diabetes. Further research is required to elucidate the mechanisms underlying these associations.

## 1 Introduction

Persistent inflammation of the sinuses, known as chronic rhinosinusitis (CRS), persists for over three months (1). To confirm a diagnosis, patients must exhibit a minimum of two of the following symptoms: nasal blockage or stuffiness, discharge from the nose either at the front or back, reduced or lost sense of smell, or discomfort/pressure/fullness in the face (2, 3). Confirmation through endoscopic or imaging findings is necessary because CRS is closely associated with a high incidence of chronic sinusitis. The refractory nature of CRS results in high treatment costs (4). Therefore, CRS is a public health concern with a substantial socioeconomic impact (5).

Diseases of autoimmune origin, including type 1 diabetes, systemic lupus erythematosus, and rheumatoid arthritis, form a category of disorders that exhibit both commonalities and distinctions. Such disorders are marked by persistent, systemic, and heightened immune responses and inflammatory processes that impact diverse tissue types throughout the body (6).

Current research has confirmed a pathophysiological association between chronic rhinosinusitis and autoimmune diseases: Autoimmune diseases may lead to the immune system attacking its own tissue, resulting in damage to the nerve endings of the sinus mucosa and the release of neuropeptide substances, thereby triggering chronic rhinosinusitis (7, 8). In patients with chronic rhinosinusitis and autoimmune diseases, there is an imbalance of inflammatory mediators, characterized by a dysregulation of the ratio between pro-inflammatory and anti-inflammatory factors, which sustains the inflammatory response (9). In individuals with autoimmune diseases, there is an abnormality in the number and function of immune cells such as T cells and B cells, which release IL-4, IL-5, IL-13, and numerous other inflammatory mediators, leading to impaired barrier function of the sinus mucosa, making it easier for pathogenic microorganisms to invade and induce infection and inflammation (10).

Large-scale prospective and retrospective studies have identified a bidirectional association between CRS and autoimmune diseases (11–19). Lately, a variety of research endeavors have shown the potency of pharmaceutical therapies for autoimmune disorders, including but not limited to CRS, nasal polyps, and comparable ailments, as shown in recent literature (20–23). However, convincing and methodical proof that establishes a connection among these conditions is still scarce.

Clarifying the linkages within large-scale conventional demographic studies proves difficult due to the influence of unnoticed intervening or influencing variables. Shared genetic information between CRS and autoimmune diseases may provide valuable insights into their correlation. Genetic links present unique benefits compared to observational studies because they are immune to traditional confounders or reverse causation, thanks to the inherent randomness in the distribution of genes during conception references (24–26).

This study aimed to analyze the causal relationship between CRS and autoimmune diseases. By leveraging genetic associations, we can overcome the limitations associated with confounding biases and provide a more robust understanding of the interplay between these conditions.

## 2 Manuscript

### 2.1 Data

The CRS GWAS data and other autoimmune diseases were retrieved from ieuOpenGWAS (https://gwas.mrcieu.ac.uk/), the FinnGen alliance (https://r8.finngen.fi/), the UK Biobank (https://www.ukbiobank.ac.uk/), and the EBI database (https://www.ebi.ac.uk/).

Summary of GWAS statistics for chronic rhinosinusitis, individual autoimmune phenotypes, and clinical traits were obtained from publicly available sources. The characteristics and sources of the SNPs instruments are listed in Table 1.

**Table 1:** Investigated genome-wide association study data sets.

Trait	Data source	Year	Cases	Controls	Sample size	IEU-ID	EXP/OUT
CRS	Neale lab	2018	1,179	360,015	361,194	ukb-d-J32	EXP 1e-5 LDSC
	FinnGen project	2021	8,524	167,849	176,373	finn-b-J10_CHRONSINUSITIS	OUT
Autoimmune diseases	FinnGen project	2021	42,202	176,590	218,792	finn-b-AUTOIMMUNE	EXP/OUT 5e-8
Type 1 diabetes	Onengut-Gumuscu et al. (27)	2015	6,683	1,217 3	29,652	ebi-a-GCST005536	EXP 5e-8 LDSC
	FinnGen project	2020	9,266	15,574	24,840	finn-b-T1D_STRICT	OUT
Systemic lupus erythematosus	FinnGen project	2021	538	213,145	213,683	finn-b-M13_SLE	Exp/out 1e-5
hypothyroidism/myxoedema	UK Biobank	2017	16,376	320,783	337,159	ukb-a-77	EXP 5e-8 LDSC
Ankylosing spondylitis	UK Biobank	2018	1,296	461,637	462,933	ukb-b-18194	EXP 5e-8
	FinnGen project	2021	1,462	164,682	166,144	finn-b-M13_ANKYLOSPON	OUT
Rheumatoid arthritis	Neale Lab	2018	1,605	359,589	361,194	ukb-d-M13_RHEUMA	EXP 1e-5 LDSC
	Okada et al. (28)	2014	19,234	61,565	80,799	ieu-a-833	OUT
Crohn's disease	De Lange et al. (29)	2017	12,194	28,072	40,266	ebi-a-GCST004132	EXP/OUT 5e-8
Ulcerative colitis	De.Lange et al. (29)	2018	12,366	33,609	45,975	ebi-a-GCST004133	EXP/OUT 5e-8
Asthma	Ben Elsworth	2018	53,257	408,756	462,013	ukb-b-20296	EXP 5e-8 LDSC
	FinnGen project	2018	4,859	135,449	140,308	finn-b-ALLERG_ASTHMA	Metal 5e-8
Allergic rhinitis	UK Biobank	2018	26,107	436,826	7,462,933	ukb-b-16499	EXP 5e-8 LDSC
	FinnGen project	2021	5,527	212,387	217,914	finn-b-ALLERG_RHINITIS	OUT
Psoriasis	UK Biobank	2018	5,314	457,619	462,933	ukb-b-10537	EXP 5e-8 LDSC
	FinnGen project	2021	4,510	212,242	216,752	finn-b-L12_PSORIASIS	OUT
Feeling nervous	Nagel et al. (30)	2018	NA	NA	373,121	ebi-a-GCST006948	MVMR
Worry	Nagel et al. (31)	2018	NA	NA	348,219	ebi-a-GCST006478	MVMR
Smoke	Ben Elsworth	2018	280,508	180,558	461,066	ukb-b-20261	MVMR
BMI	Ben Elsworth	2018	NA	461,460	NA	ukb-b-19953	MVMR

Data source used to perform Mendelian randomization analysis in this study.

Informed consent was obtained from all participants in each study, and ethical approval was obtained from the respective institutional review boards. We comprehensively screened the literature to test for horizontal pleiotropy in the selected SNPs and assessed whether any SNPs were affected by linkage disequilibrium (LD) (Table 1).

### 2.2 Methods

**Figure 1:**
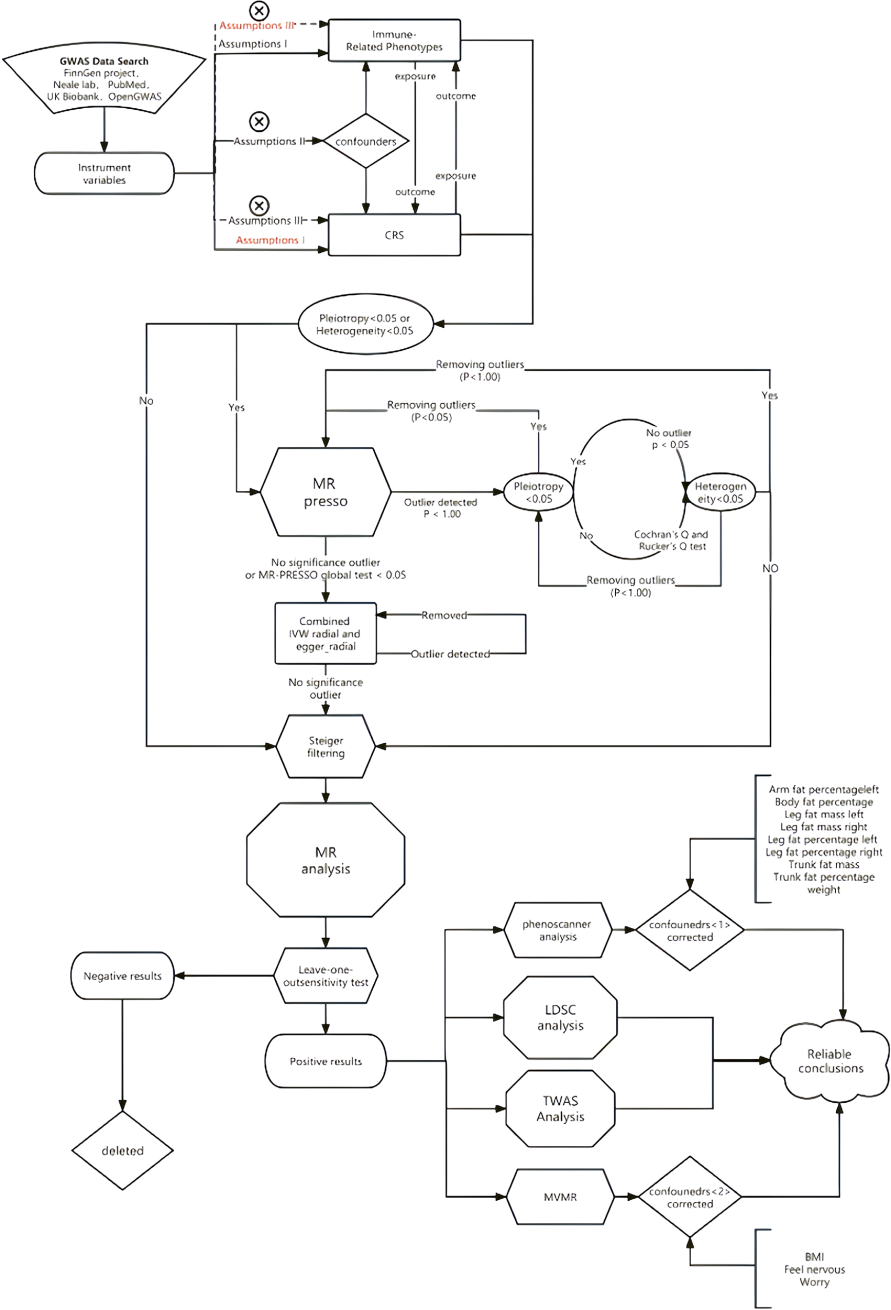
The specific methodological workflow.

We employed two-sample Mendelian Randomization, Multivariate Mendelian Randomization (MVMR), LDSC, and TWAS to assess the association between chronic sinusitis and autoimmune diseases. Additionally, we used MR-PRESSO and RadialMR to correct for horizontal pleiotropy and heterogeneity in the MR results. The specific methodological workflow was as follows (Figure 1).

#### 2.2.1 Introduction to the principle of MR

MR is a widely used analytical approach in which genetic variants act as instrumental variables and genetic predictors serve as independent risk factors. These genetic variants are naturally randomized during meiosis, resulting in an independent distribution within the population. Consequently, the MR is theoretically immune to potential environmental confounders and disease states. There are three fundamental assumptions underlying MR: (1) genetic variants are associated with risk factors (association hypothesis), (2) these genetic variants exhibit no association with any known or unknown confounders (independence assumption), and (3) genetic variation solely influences outcomes through risk factors (exclusion restriction hypothesis) (23).

The third assumption, also known as pleiotropy without a direct effect on outcomes, occurs when genetic variants impact outcomes through alternative pathways independent of risk factors. We used a comprehensive range of methods and sensitivity tests to assess these assumptions. To evaluate hypotheses (2) and (3), we selected the sample with the lowest *p*-value associated with clinical outcomes.

#### 2.2.2 Two-sample MR

We used classical two-sample MR as a screening method for genetic associations to describe multiple bidirectional two-sample MR studies and to estimate the causal effects, including CRS and type 1 diabetes mellitus, allergic rhinitis, rheumatoid arthritis, systemic lupus erythematosus, Crohn's disease, ulcerative colitishypothyroidism, and asthma. We employed various databases as exposures or outcomes for the same disease (as indicated in the document), and conducted a two-way Mendelian randomization analysis for the two conditions.

The analysis was performed using the software package two- sample MR with R 4.2.1, using a genome-wide threshold of *p* < 5 × 10-8,r2 < 0.1 to define the genetic instrument, which we relaxed to *p* < 1 × 10-5 in the case of fewer than 15 SNPs.

#### 2.2.3 MR-PRESSO

The approach for removing outliers was as follows: First, an assessment was made to determine the presence of pleiotropy and heterogeneity(*p* < 0.05) between exposure and outcome 24. The MR-PRESSO test was conducted as an initial step in cases where pleiotropy and/or heterogeneity were detected. Based on the results of the MR-PRESSO test, a decision was made regarding the removal of outliers and subsequent assessments of pleiotropy and heterogeneity were performed after outlier removal. If positive results persisted, the aforementioned steps were repeated until no pleiotropy or heterogeneity remained. If there was no evidence of pleiotropy or heterogeneity between the original exposure and outcome, the MR-PRESSO test was not performed and a directional test was conducted to identify and exclude outliers that exhibited an opposite causal relationship.

#### 2.2.4 MR methods

For the analysis, we employed five commonly used MR analysis methods: (MR Egger, inverse variance weighted, weighted median, simple mode, and weighted mode). Compared with the WME, ME, and MER methods, the IVW method demonstrated greater power. A significance level of *p* < 0.05 was utilized to determine positive results using the IVW method. Additionally, we used the mr_MaxLik, mr_mbe, and mr_Median methods to provide supplementary evidence for our results. Finally, left-one-out analysis was conducted to evaluate whether any single SNP disproportionately influenced the association.

#### 2.2.5 LDSC analysis

We conducted LDSC analysis to investigate the genetic associations between hypothyroidism, rheumatoid arthritis, type 1 diabetes, allergic rhinitis, asthma, and chronic rhinosinusitis. The LDSC was used to estimate heritability and assess genetic associations based on single nucleotide variants. LDSC regression was employed to evaluate the genetic association between all autoimmune diseases and CRS, considering single nucleotide variants(SNV) heritability (h2) and the magnitude of correlation (rg) as indicators.

#### 2.2.6 Phenoscanner

To ensure robustness and account for potential confounding factors, we employed PhenoScanner 25 (www.phenoscanner.medschl.cam.ac.uk) to verify the instruments used in the analysis and their association with autoimmune diseases and CRS. Additionally, we re-validated the results to ascertain whether the correlations were strengthened after eliminating confounders.

#### 2.2.7 MVMR analysis

Simultaneously, we conducted MVMR to identify and adjust for weak instrumental variables in the two-sample estimation. Using two-sample MR 26, we explored the risk factors associated with CRS, revealing that smoking, occupational environmental dust content, anxiety, stress, and other emotional changes were significant contributors to the incidence of chronic rhinosinusitis. To further account for potential confounders and examine the impact of the PhenoScanner correction, we designed a multigroup MVMR analysis. Specifically, we used Type 1 diabetes mellitus (MVMR1), hypothyroidism (MVMR2), asthma (MVMR3), allergic rhinitis (MVMR4), and rheumatoid arthritis (MVMR5) as covariates in the analysis.

#### 2.2.8 TWAS analysis

To analyze the genetic mechanisms shared between autoimmune phenotypes and chronic rhinosinusitis, we performed TWAS. Initially, we generated GWAS summary statistics for CRS and autoimmune diseases using the sumstats.py script provided by LDSC. FUSION is a suite of tools for performing transcriptome-wide and regulome-wide association studies. Following that, we utilized the FUSION pipeline in its preset mode to calculate the transcriptomic profiles linked to each respective outcome. Through this application of FUSION, cross-tissue cis-genetic markers of gene expression were detected (32). These signatures were used, along with our gene expression weights, to construct linear predictors for the SNP-based expression levels of each cis-genetic signature. The TWAS test statistics were then calculated based on linear predictors and summary GWAS z-scores. This allowed us to further investigate the shared genetic mechanisms underlying autoimmune phenotypes and chronic rhinosinusitis.

Single-trait TWAS Analysis We employed a threshold of PPH3 + PPH4 > 0.8 as the criterion for identifying co-localization of significant genes, assessing the mapping relationship between each gene and trait. Proceeding to the gene level, we investigated the existence of gene expression associated with chronic sinusitis and autoimmune diseases. The findings from TWAS indicate a considerable degree of shared significant correlations within organs including the nervous, endocrine, cardiovascular, and gastrointestinal systems, reinforcing the idea that the intricate regulatory nexus between chronic sinusitis and traits of autoimmune diseases is multifaceted.

### 2.3 Results

#### 2.3.1 MR results

Bivariate bidirectional MR was employed to construct multiple groups of exposures and outcomes for MR analysis, which revealed significant associations with various autoimmune conditions. Our findings indicate that the risk of AR (*p* = 9.55E-10, OR = 2,711.019278702), asthma (*p* = 1.81E-23, OR = 33.9964343413), rheumatoid arthritis (*p* = 9.55E-10, OR = 1.11552606451696), hypothyroidism (*p* = 0.0208828, OR = 4.84925374492983), and Type 1 diabetes (*p* = 0.0208828, OR = 1.04848968536672) is genetically influenced (Table 2).

**Table 2:** The results of LDSC analysis and MR analysis.

Exposure	Outcome	LDSC analysis		MR analysis				
		rg	*P* val*	Method	*P* val*	or	or lci95	or uci95
Type 1 diabetes	CRS	0.263510473	0-273648571	IVW	0.0208828	1.04849	1.016293	1.081706
RA	CRS	0 27834931	0.035376	IVW	9.55E-10	1.115526	1.079948	1.152276
AR	CRS	0.344724754	0.000000394	IVW	9.55E-10	2711.019 261.83391		28069.8
AT	CRS	0.43703672	1.86E-10	IVW	1 81E-23	33.99643	17.52439	65.95137
Hypothyroidism/myxoedema	CRS	0.213201473	0.000383093	IVW	0.020882848	4.849254	1.715446	13.70796

*B-H corrected.

#### 2.3.2 LDSC results

Furthermore, LDSC analysis revealed a significant genetic association between each autoimmune phenotype and CRS. Specifically, AR demonstrated a strong correlation (rg = 0.344724754, *p* = 0.000000394), as did asthma (rg = 0.43703672, *p* = 1.86E-10), rheumatoid arthritis (rg = 0.27834931, *p* = 0.035376), and hypothyroidism (rg = −0.213201473, *p* = 0.000383093). Type 1 diabetes also showed a moderate genetic association with CRS (rg = 0.263510473, *p* = 0.273648571).

These results corroborate the theory suggesting a genetic overlap between these autoimmune disorders and CRS, thereby emphasizing their possible involvement in the onset and progression of this concurrent condition.

**Figure 2:**
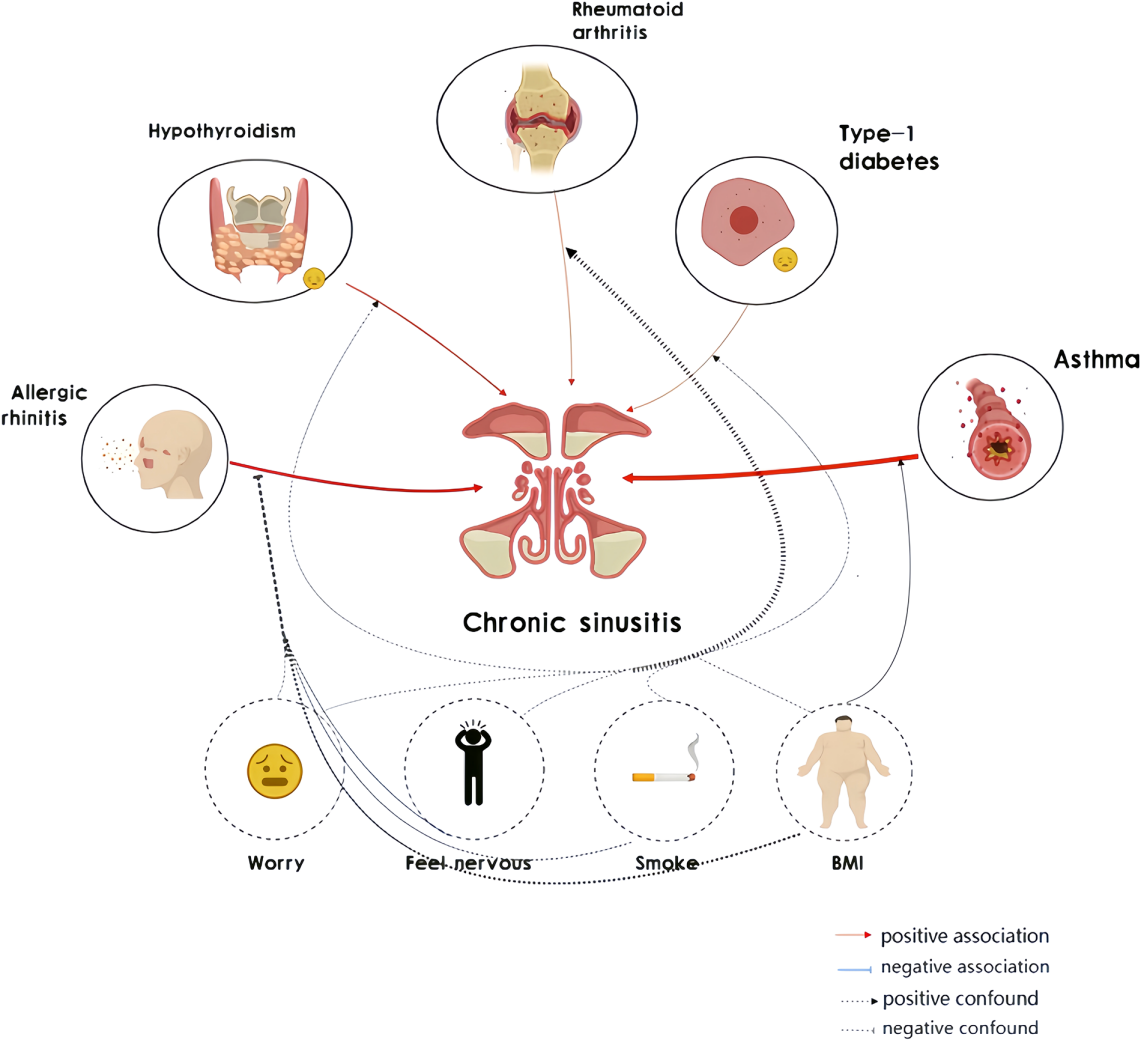
Mendelian randomization (MR) associations linking chronic sinusitis and autoimmune diseases after adjustment for other risk factors.

Through PhenoScanner analysis, associations were identified between certain SNPs and chronic sinusitis. Notably, these SNPs were also associated with confounding factors such as fat distribution and body weight. In response to potential sources of confusion, a subsequent magnetic resonance examination was carried out post-adjustment for such elements. Post-adjustment, the link between asthma, hypothyroidism, and chronic sinusitis was found to be more pronounced. Additionally, body mass index (BMI) was considered a variable in the study due to its potential role in influencing this relationship (Figure 2).

#### 2.3.3 MVMR results

**Figure 3:**
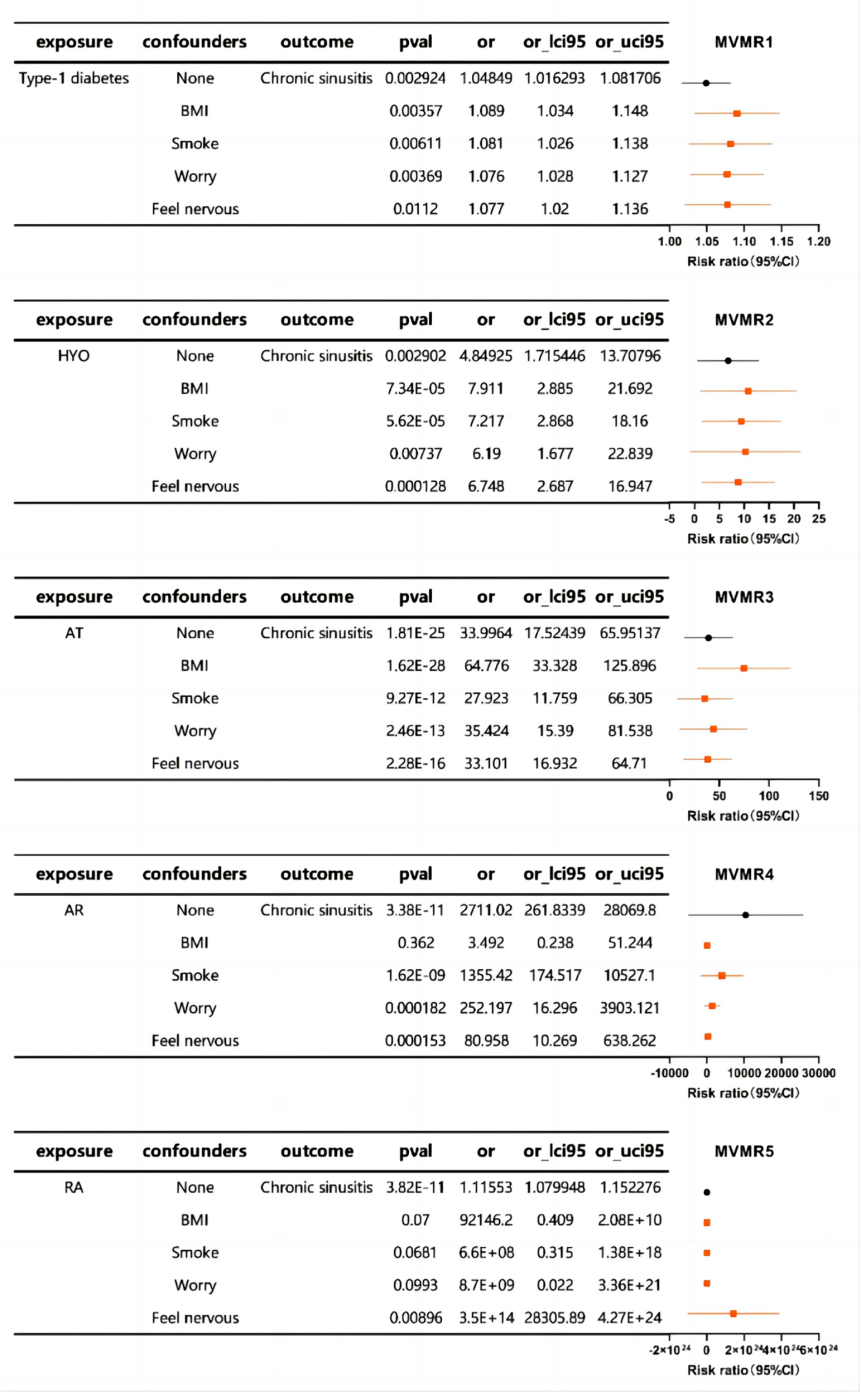
The forest plot of MVMR exploring the associations between autoimmune diseases and chronic sinusitis adjusted for confounding traits.

Subsequently, MVMR analysis was performed to explore the relationship between autoimmune diseases and chronic sinusitis in the presence of additional confounding factors. The MVMR analysis revealed associations between BMI, smoking behavior, and mood factors, as well as an association between autoimmune diseases and chronic sinusitis. This implies that such interfering elements might be intermediary variables that influence the link between the immunological profiles and sinusitis to some extent (Figure 3).

In essence, our research underscores the significance of accounting for confounders in genetic research on chronic sinusitis and points to the possible intermediary influence of elements like body mass index, smoking habits, and emotional state in the nexus between autoimmune disorders and chronic sinusitis.

#### 2.3.4 TWAS results

**Figure 4:**
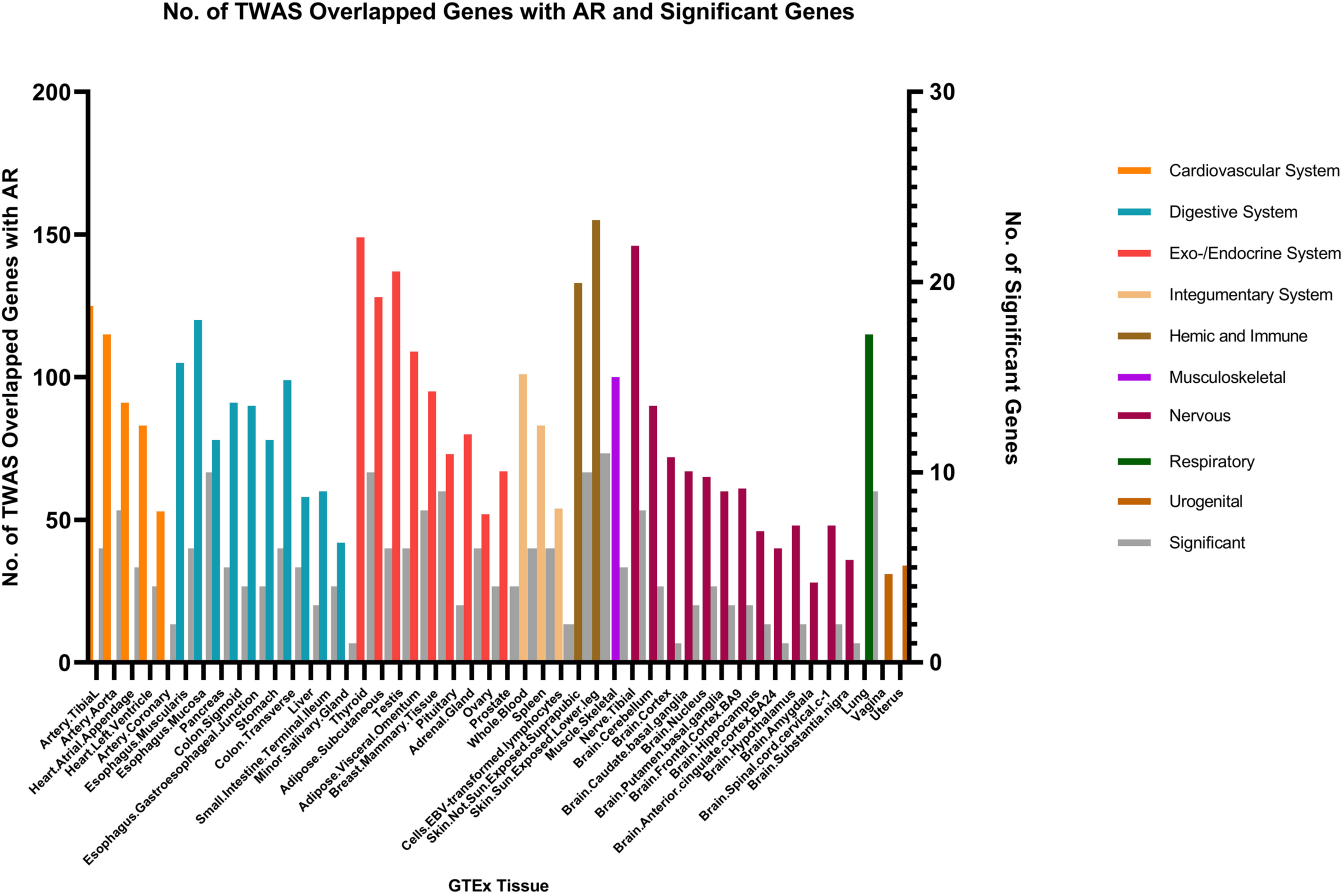
The result of transcriptome-wide association (TWAS) analysis of AR.

We identified multiple genes shared between chronic sinusitis and AR, predominantly located within the endocrine/exocrine system (149 genes in thyroid tissue, 137 in testes, and 128 in subcutaneous tissue), digestive system (120 genes in esophageal mucosa and 105 in esophageal muscular layer), skin (288 shared genes), and nervous system (146 shared genes in tibial nerve) tissues. Notably, the skin tissue exhibited a relatively higher number of key shared genes (Figure 4).

**Figure 5:**
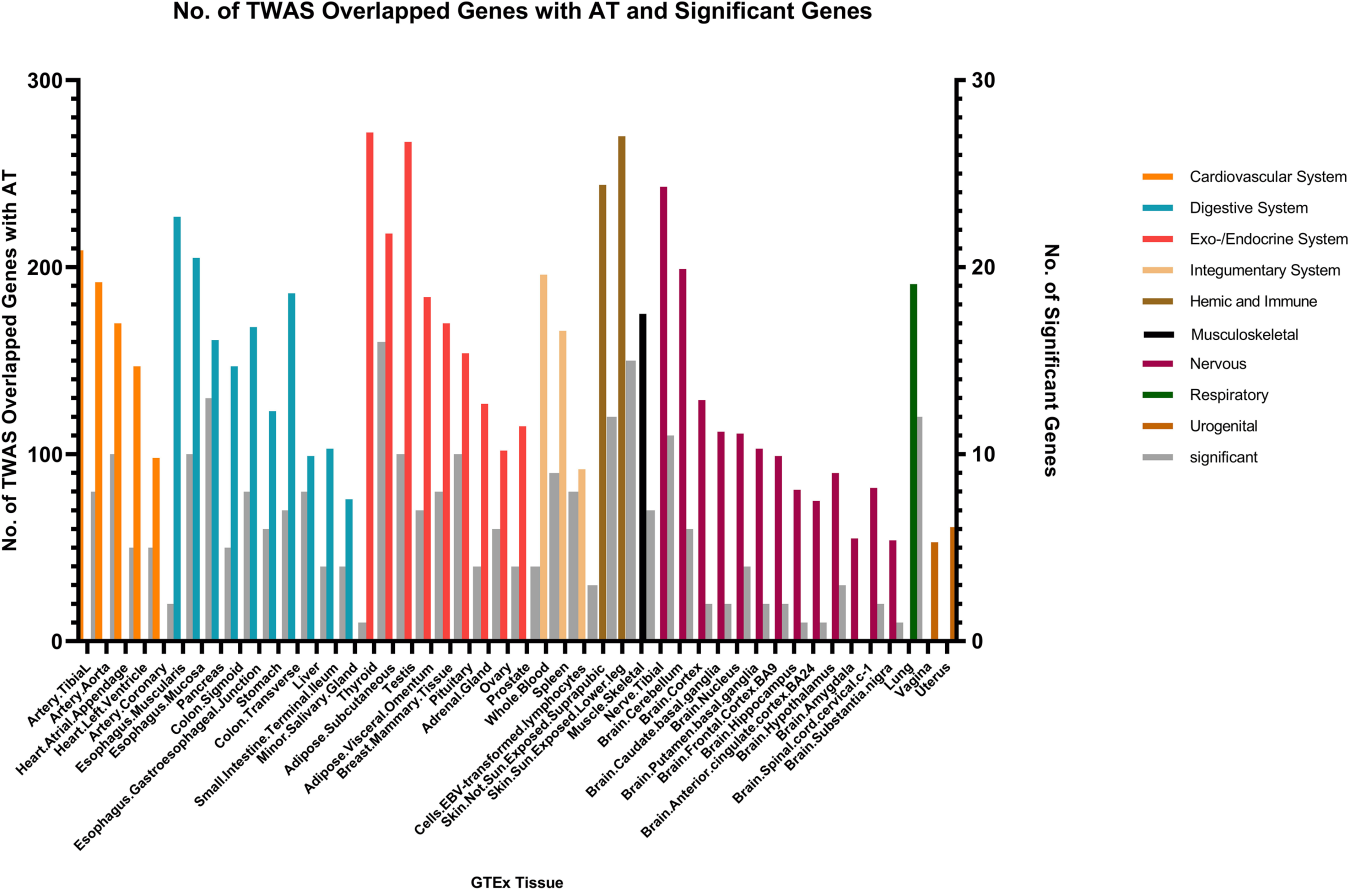
The result of transcriptome-wide association (TWAS) analysis of AT.

In the context of AT and CRS, numerous genes were shared across various tissues, with the endocrine/exocrine system showing the highest distribution. Genes such as TSLP/WDR36 (CHR5, Best SNP: rs1837253), ORMDL3(CHR13, Best SNP: rs11557467), and IL1RL1/IL18R1(CHR2,Best SNP: rs12905) demonstrated a higher degree of concordance in sharing between AT, AR, and CRS, suggesting a relatively stronger genetic association among these traits compared to other autoimmune diseases (Figure 5).

**Figure 6:**
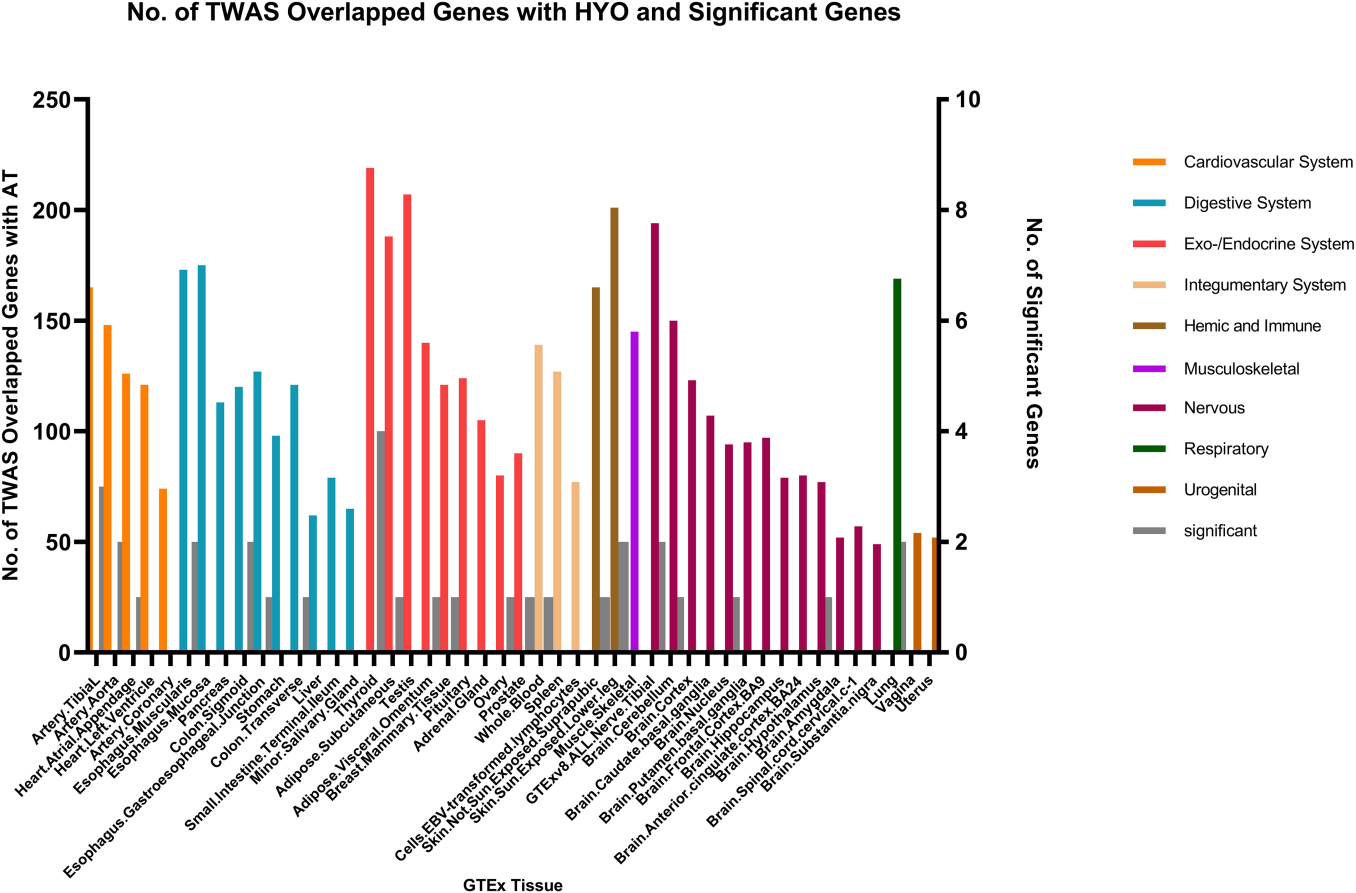
The result of transcriptome-wide association (TWAS) analysis of HYO.

**Figure 7:**
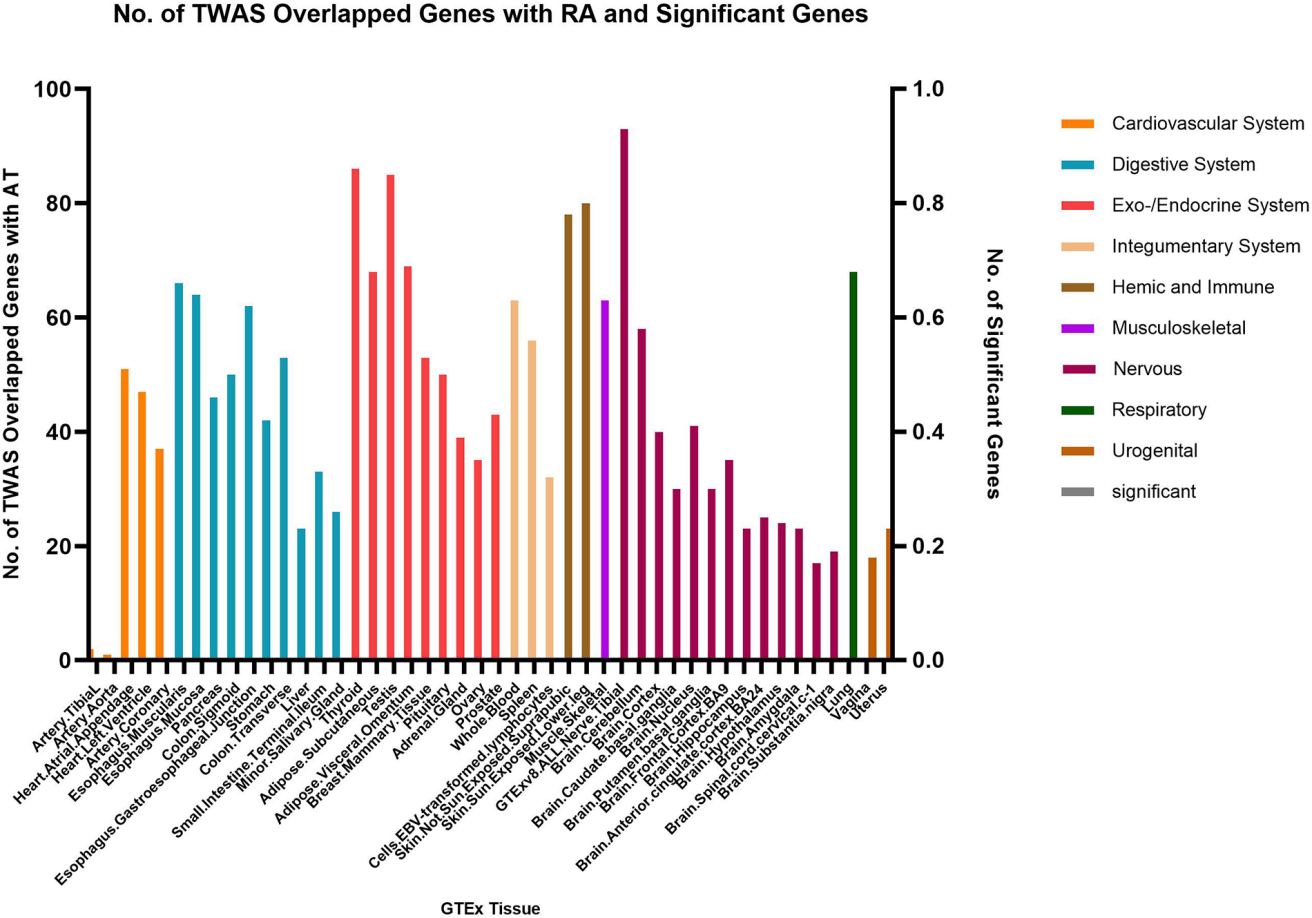
The result of transcriptome-wide association (TWAS) analysis of RA.

**Figure 8:**
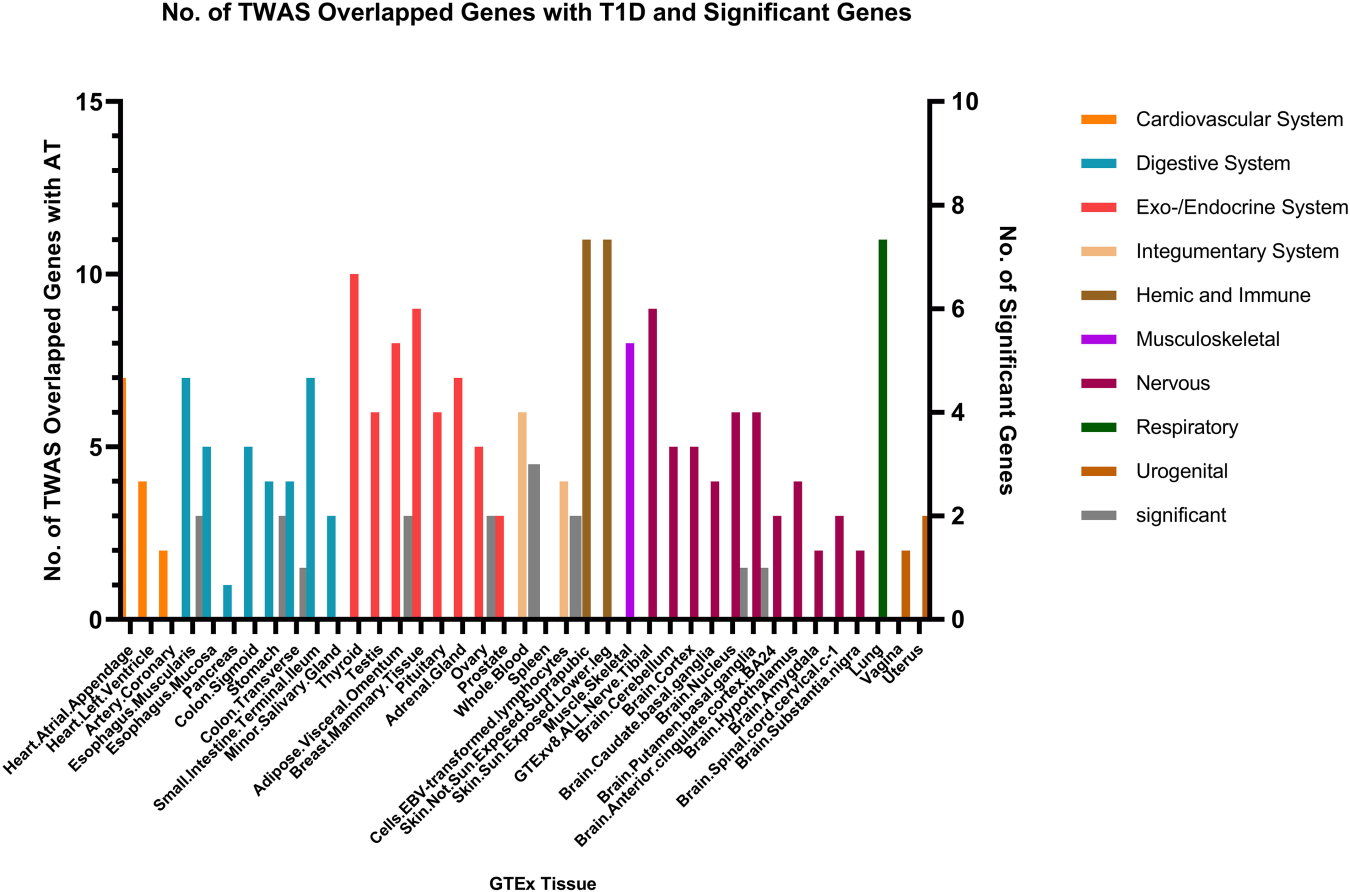
The result of transcriptome-wide association (TWAS) analysis of T1D.

Fewer shared genes were identified between RA, HYO, T1D, and CRS. PSMB7 was found to be a shared gene between CRS and HYO in multiple tissues, including the cardiovascular system (atrial and aortic tissue), digestive system (stomach and esophageal mucosa), and endocrine system (subcutaneous fat, oral, and mammary gland). PGAP3 was identified as a shared gene between CRS and T1D in tissues such as esophageal mucosa, transverse colon, and brain. To date, no significant shared genes have been found between RA and CRS (Figures 6–8).

## 3 Discussion

In this genetic association study, shared genetic liability across the genome was quantified, encompassing various chronic sinusitis and immunophenotypes, along with their associated risk factors.

Genetic links exist between CRS and autoimmune manifestations like allergic rhinitis, asthma, rheumatoid arthritis, hypothyroidism, and type 1 diabetes. Furthermore, we validated conventional risk factors for CRS, including smoking, dust exposure in the workplace, and mood changes, and assessed their impact on the causal relationship between autoimmune phenotypes and CRS.

### 3.1 Allergic rhinitis, asthma, rheumatoid arthritis, type 1 diabetes, hypothyroidism and CRS

#### 3.1.1 Genetic association

Persistent forms of rhinosinusitis, allergic nasal inflammation, and skin conditions characterized by atopy demonstrate a marked coherence in their expression characteristics linked to the TSLP gene located on the fifth chromosome. The TSLP gene is responsible for producing a protein that facilitates the activation of Type 2 T helper (TH2) cell activities and plays a role in the initiation and advancement of numerous chronic inflammatory disorders, a conclusion that is in agreement with the research presented by Hong and colleagues (33). TSLP has been shown to modulate the production of eotaxin-1 by nasal epithelial cells in eosinophilic CRS with nasal polyps (CRSwNP), thereby facilitating the development of eosinophilic sinus inflammation (34) and TSLP plays a critical role in initiating allergen sensitization (35). Our study suggests that TSLP may serve as a common genetic mechanism underlying sinusitis and autoimmune diseases.

ORMDL3 represents a shared genetic component between CRS, AR, and AT as well. OEMDL3 serves as a significant indicator for asthma and contributes to the development of multiple inflammatory disorders. Imbalance in ceramide levels may worsen the manifestations of allergic asthma and associated ailments (36, 37). It exacerbates symptoms of allergic asthma and other diseases by disrupting the ceramide homeostasis (38). However, no current research has established a correlation between ORMDL3 and CRS. Our study suggests that ORMDL3 may serve as a genetic marker for CRS, a hypothesis that requires further study for validation.

Previous research has associated IL1RL1 and IL18R1 with asthma and other atopic diseases (39). The Castano R team found that the expression of IL1RL1 is related to chronic rhinosinusitis (40). Our study confirms that these genes are expressed in multiple tissues of patients with CRS, AT, and AR, further indicating a potential genetic basis shared among the three conditions.

WDR36 is co-expressed in multiple tissues of patients with chronic sinusitis, asthma, and allergic rhinitis. It has been identified as a locus for eosinophilic esophagitis (41); however, no research has linked WDR36 to CRS. Our study suggests a correlation between TSLP, WDR36 and CRS, AT, and AR.

Our research reveals that the PGAP3 gene is expressed in multiple tissues of patients with CRS, AR, AT, and type 1 diabetes, indicating it may serve as a common genetic basis for chronic sinusitis and autoimmune diseases. This finding is consistent with the conclusions of the Zerun Song team (42) and the Cristina T Vicente team (39).

We found that PSMB7 is expressed in multiple tissues of CRS and HYO patients, and GLDC is expressed in multiple tissues of CRS, AT, and AR patients. Although no current research has established a link between PSMB7 and these conditions, our study suggests that these genes may be potential genetic markers for the common genetic basis of CRS and autoimmune diseases.

GSDMB, a diagnostic gene for type 1 diabetes (43), actively participates in the onset and progression of metabolic disorders by influencing body fat distribution (44). Moreover, it regulates genetic activity via targeted methylation of DNA, playing a role in the progression of autoimmune conditions such as rheumatoid arthritis and asthma, among others, as noted in reference (45). Some studies have also revealed a relationship between GSDMB and chronic sinusitis (46).

In line with prior research, our investigation corroborated that GSDMB is a common genetic link between the trio of ailments. This discovery provides a valuable basis for the identification of therapeutic targets for autoimmune diseases and chronic rhinosinusitis. Using MR analysis, our study suggested that rheumatoid arthritis and type 1 diabetes mellitus are risk factors for chronic rhinosinusitis. Nevertheless, further research is required to clarify the causal relationship linking these three disorders.

#### 3.1.2 Biochemical correlation

Th-17 cells are a critical type of immune cell that play a pivotal role in the development and progression of autoimmune diseases and chronic rhinosinusitis (CRS). These cells primarily secrete cytokines such as interleukin (IL)-17 and IL-22, which are essential in regulating immune responses and maintaining tissue homeostasis. However, the abnormal activation of Th-17 cells can lead to the onset of autoimmune diseases (47, 48). Cytokines like IL-17 can promote the recruitment and activation of inflammatory cells, resulting in tissue damage. In autoimmune diseases such as rheumatoid arthritis and multiple sclerosis, the levels of Th-17 cells and their secreted cytokines are typically elevated. Th-17 cells may be involved in abnormal immune responses against self-antigens, leading to an attack on the body's own tissues and thereby triggering autoimmune diseases.

In patients with chronic rhinosinusitis, the expression of Th-17 cells and their secreted cytokine IL-17A is upregulated in the sinus mucosa, leading to intensified inflammatory responses, subsequent mucosal damage, and pathological remodeling. The imbalance between Th-17 cells and regulatory T cells (Tregs) in the bodies of chronic rhinosinusitis patients may contribute to the persistence of inflammation and the chronic nature of the disease (49).

Th-17 cells and their cytokines are involved in the same inflammatory pathways in both autoimmune diseases and chronic rhinosinusitis, such as the IL-17/IL-23 axis (50). During the progression of these diseases, Th-17 cells may act as a bridge, exacerbating the disease process.

In summary, the correlation between Th-17 cells and autoimmune diseases and chronic rhinosinusitis is primarily manifested in immune regulation, inflammation mediation, and tissue remodeling. Delving into the mechanisms of Th-17 cells in these diseases can provide new targets and methods for clinical treatment.

Recent studies on allergic respiratory diseases and sinusitis have shown that the association between these conditions may be attributed to the destruction of the epithelial barrier or the production of cytokines. The protective layer of epithelium coating the nasal mucosa acts as an innate safeguard against inflammation within the nasal cavity (51). It seems that the onset of conditions such as allergic rhinitis, asthma, and chronic rhinosinusitis is associated with a dysfunction of this epithelial defense (52).

Persistent engagement of type 2 immune responses within the upper airways may result in allergic swelling, culminating in enduring rhinosinusitis spanning a spectrum of seriousness from mild rhinitis to intense nasal polyp formation (53). A hypothesis has been put forth highlighting the significance of epithelium-derived cytokines, including IL-25, IL-33, and TSLP, as key regulators that connect epithelial-mesenchymal communication and induce pathophysiological changes in the airways (33).

Continued examination of these cytokines originating from epithelial cells is expected to provide deep understanding of the processes that drive inflammation in the respiratory tract. Consequently, employing agents that act upon these cytokines could accelerate progress in the therapy and control of allergic rhinitis, chronic sinus infections, and related disorders, thereby improving the health outcomes for individuals suffering from asthma and allergic reactions.

### 3.2 Confounding factors and CRS

Numerous research reports indicate a marginal increase in obesity measurements among individuals suffering from long-term sinus inflammation compared to the broader populace (54), implying that body mass index serves as a contributing element. Furthermore, cigarette smoke has been recognized as a contributing element to persistent sinus inflammation (55), as have psychological influences. Furthermore, our study revealed that these confounding factors were also involved in the association between autoimmune diseases and chronic rhinosinusitis (56, 57).

### 3.3 Innovations of our study design

In contrast to the traditional two-sample Mendelian randomization (MR) analysis, we employed a bidirectional causal inference approach, further mitigating the potential for horizontal pleiotropy and heterogeneity by utilizing multiple rounds of MRpresso. Additionally, we utilized linkage disequilibrium score regression (LDSC) to further assess whether there was genetic correlation among the positive MR results. Transcriptome-wide association studies (TWAS) were employed to identify common functional genes between the conditions, and we explored the potential pathophysiological processes in which these key genes might be involved. Compared to the classic two-sample MR, our study exhibits a higher level of rigor, offering genetic correlation targets between diseases, thereby providing a stronger foundation for research on disease associations.

### 3.4 Limitations of our study design

One drawback of the MR design is that it can only be applied to risk factors with suitable genetic variants. Genetic variation generally has little effect on most risk factors, they elucidate a limited range of divergences, potentially resulting in diminished statistical strength for Mendelian Randomization studies and an increased likelihood of type II errors. Additional empirical investigation is necessary to clarify how these intervening variables affect the association.

### 3.5 Future perspectives

The association between chronic rhinosinusitis (CRS) and autoimmune diseases has been increasingly revealed, and future treatment developments will focus on this connection, aiming to more effectively control the disease and improve the quality of life for patients (58).

Utilizing high-throughput sequencing, proteomics, and other technologies, the genetic and protein expressions of patients with chronic rhinosinusitis and autoimmune diseases will be examined to achieve precise diagnosis and classification of the condition. The search for common biomarkers between CRS and autoimmune diseases will provide a basis for disease monitoring, efficacy assessment, and prognostic judgment.

Targeted biologics that modulate immune cells, such as T cells and B cells, will be developed to address the immune imbalance in patients with CRS and autoimmune diseases (59). Inhibitors targeting key inflammatory mediators, such as IL-4, IL-5, and IL-13, will be created to reduce inflammation and improve the condition (60).

The study of genetic associations between chronic rhinosinusitis and autoimmune diseases has opened up the possibility for personalized treatment. Genetic association research uncovers the relationship between these conditions and specific gene variants, offering insights into early disease diagnosis and risk prediction (61). Based on a patient's genetic background, physicians can more accurately determine if they have a particular disease and predict its progression.

Furthermore, we can select targeted drugs or treatment methods based on gene mutations. Genetic studies also predict patients’ responses to different medications, thereby avoiding unnecessary drug trials. This enhances the safety and efficacy of drug therapy, ultimately increasing the success rate of treatment, reducing side effects, and conserving medical resources to provide patients with more targeted care (62).

An integrated approach that combines pharmacological treatment, surgical intervention, and traditional Chinese medicine, along with a multidisciplinary collaboration involving otolaryngology, immunology, and allergy departments, will offer comprehensive and personalized treatment plans for patients. Prevention measures targeting common risk factors for CRS and autoimmune diseases will be implemented to reduce the incidence rate and comprehensively enhance the quality of life for patients (63).

## 4 Conclusions

In conclusion, this study employed LDSC regression, MR analysis with large GWAS data, and TWAS to investigate the genetic association and causality between autoimmune phenotypes and chronic sinusitis. Our findings confirmed the association between autoimmune diseases and chronic sinusitis. The association between chronic rhinosinusitis and autoimmune diseases holds significant importance in clinical practice, providing guiding insights into the diagnostic process, treatment strategies, disease monitoring, and health education at each stage. These results have significant implications for guiding drug regimens for the treatment of chronic sinusitis.

## Data Availability

The original contributions presented in the study are included in the article/Supplementary Material, further inquiries can be directed to the corresponding author.
