# Supplementary material for: Genetic correlation between chronic sinusitis and autoimmune diseases
Source: Front Allergy. 2024 Sep 24;5:1387774. doi: 10.3389/falgy.2024.1387774 (PMC11458559; doi:10.3389/falgy.2024.1387774)
Supplement: Supplementary file 1 [file Datasheet1.pdf]

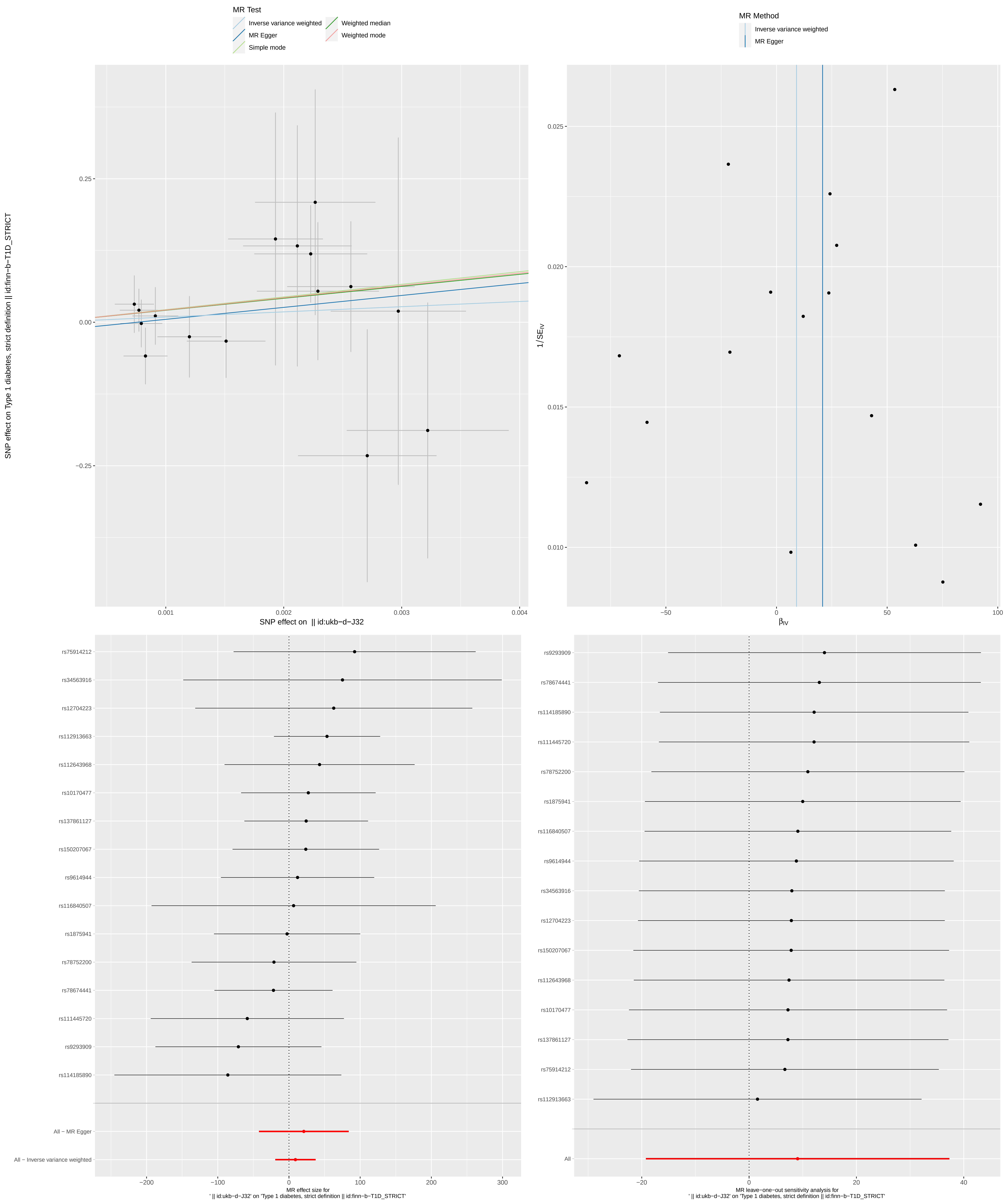
MR Test
Inverse variance weighted
MR Egger
Simple mode
Weighted median
Weighted mode
SNP effect on Type 1 diabetes, strict definition || id:finn−b−T1D_STRICT
SNP effect on || id:ukb−d−J32
0.25
0.00
−0.25
0.001
0.002
0.003
0.004
MR Method
Inverse variance weighted
MR Egger
1/SE_IV
β_IV
0.025
0.020
0.015
0.010
−50
0
50
100
rs75914212
rs34563916
rs12704223
rs112913663
rs112643968
rs10170477
rs137861127
rs150207067
rs9614944
rs116840507
rs1875941
rs78752200
rs78674441
rs111445720
rs9293909
rs114185890
All − MR Egger
All − Inverse variance weighted
−200
−100
0
100
200
300
MR effect size for
' || id:ukb−d−J32' on 'Type 1 diabetes, strict definition || id:finn−b−T1D_STRICT'
rs9293909
rs78674441
rs114185890
rs111445720
rs78752200
rs1875941
rs116840507
rs9614944
rs34563916
rs12704223
rs150207067
rs112643968
rs10170477
rs137861127
rs75914212
rs112913663
All
−20
0
20
40
MR leave−one−out sensitivity analysis for
' || id:ukb−d−J32' on 'Type 1 diabetes, strict definition || id:finn−b−T1D_STRICT'

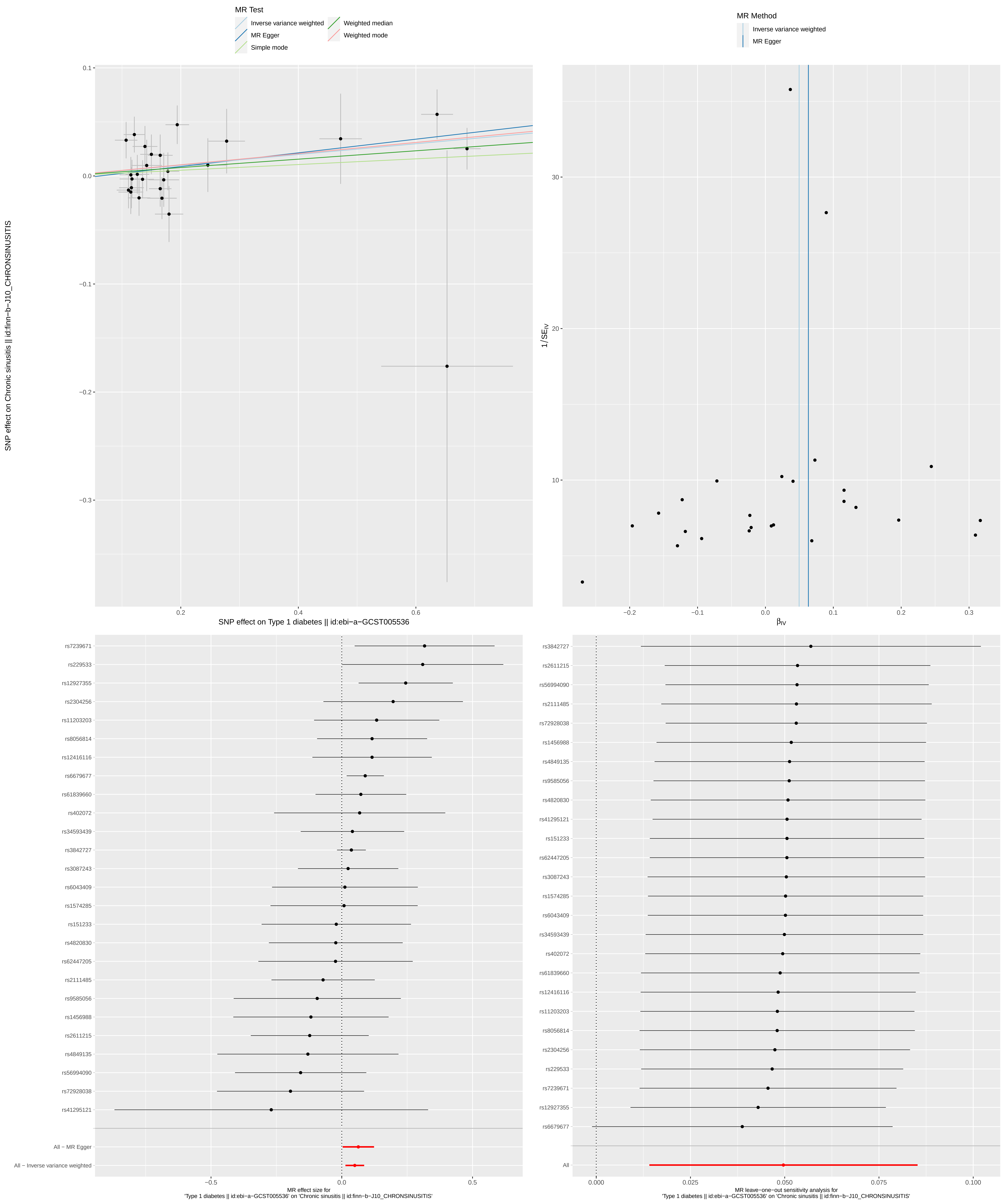

MR Test
Inverse variance weighted
MR Egger
Simple mode
Weighted median
Weighted mode
SNP effect on Chronic sinusitis || id:finn−b−J10_CHRONSINUSITIS
SNP effect on Type 1 diabetes || id:ebi−a−GCST005536
MR Method
Inverse variance weighted
MR Egger
1/SE_IV
β_IV
rs7239671
rs229533
rs12927355
rs2304256
rs11203203
rs8056814
rs12416116
rs6679677
rs61839660
rs402072
rs34593439
rs3842727
rs3087243
rs6043409
rs1574285
rs151233
rs4820830
rs62447205
rs2111485
rs9585056
rs1456988
rs2611215
rs4849135
rs56994090
rs72928038
rs41295121
All − MR Egger
All − Inverse variance weighted
MR effect size for
'Type 1 diabetes || id:ebi−a−GCST005536' on 'Chronic sinusitis || id:finn−b−J10_CHRONSINUSITIS'
rs3842727
rs2611215
rs56994090
rs2111485
rs72928038
rs1456988
rs4849135
rs9585056
rs4820830
rs41295121
rs151233
rs62447205
rs3087243
rs1574285
rs6043409
rs34593439
rs402072
rs61839660
rs12416116
rs11203203
rs8056814
rs2304256
rs229533
rs7239671
rs12927355
rs6679677
All
MR leave−one−out sensitivity analysis for
'Type 1 diabetes || id:ebi−a−GCST005536' on 'Chronic sinusitis || id:finn−b−J10_CHRONSINUSITIS'

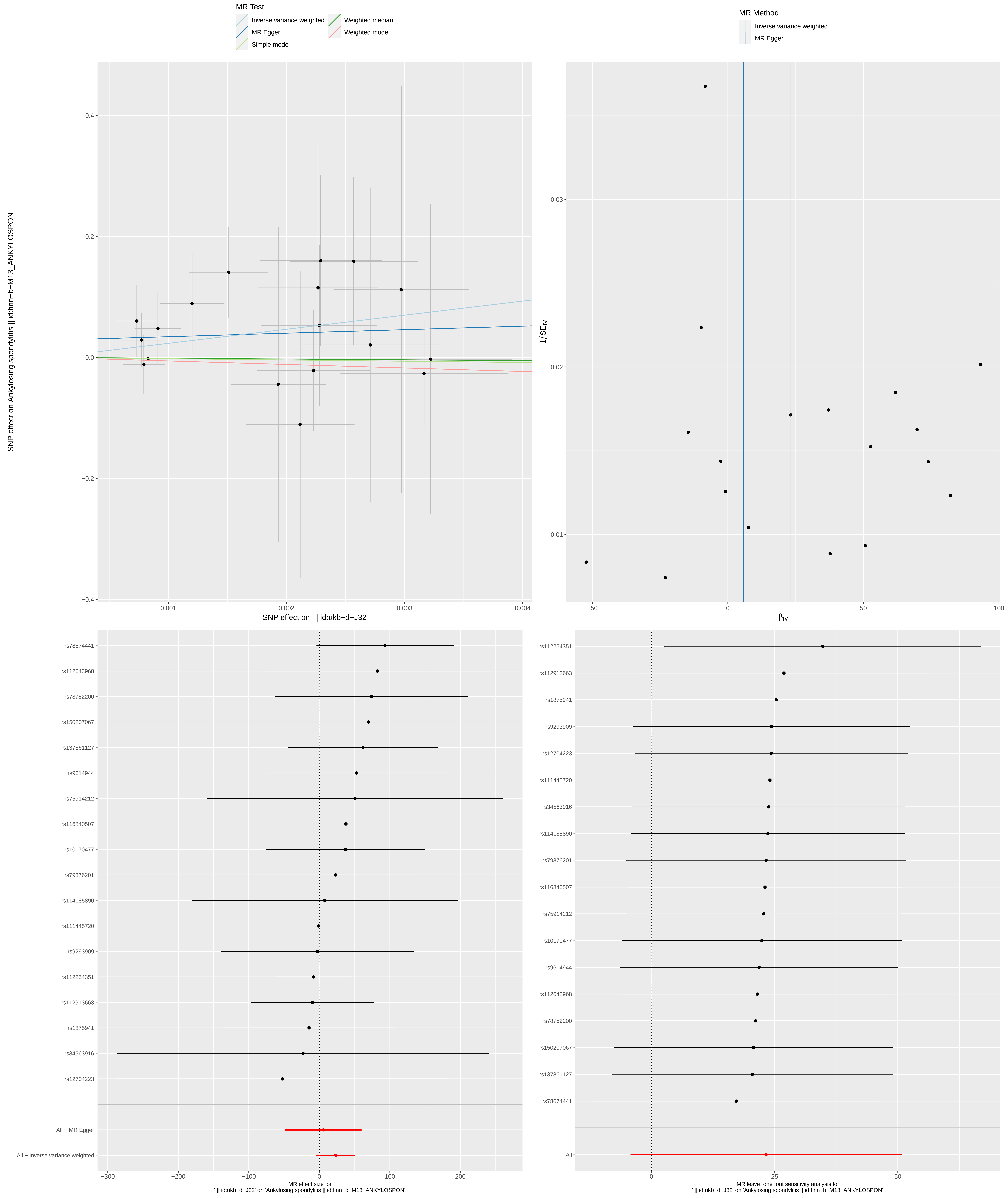
MR Test
Inverse variance weighted
MR Egger
Simple mode
Weighted median
Weighted mode
SNP effect on Ankylosing spondylitis || id:finn−b−M13_ANKYLOSPON
SNP effect on || id:ukb−d−J32
0.4
0.2
0.0
−0.2
−0.4
0.001
0.002
0.003
0.004
MR Method
Inverse variance weighted
MR Egger
1/SE_IV
β_IV
0.03
0.02
0.01
−50
0
50
100
rs78674441
rs112643968
rs78752200
rs150207067
rs137861127
rs9614944
rs75914212
rs116840507
rs10170477
rs79376201
rs114185890
rs111445720
rs9293909
rs112254351
rs112913663
rs1875941
rs34563916
rs12704223
All − MR Egger
All − Inverse variance weighted
−300
−200
−100
0
100
200
MR effect size for
' || id:ukb−d−J32' on 'Ankylosing spondylitis || id:finn−b−M13_ANKYLOSPON'
rs112254351
rs112913663
rs1875941
rs9293909
rs12704223
rs111445720
rs34563916
rs114185890
rs79376201
rs116840507
rs75914212
rs10170477
rs9614944
rs112643968
rs78752200
rs150207067
rs137861127
rs78674441
All
0
25
50
MR leave−one−out sensitivity analysis for
' || id:ukb−d−J32' on 'Ankylosing spondylitis || id:finn−b−M13_ANKYLOSPON'

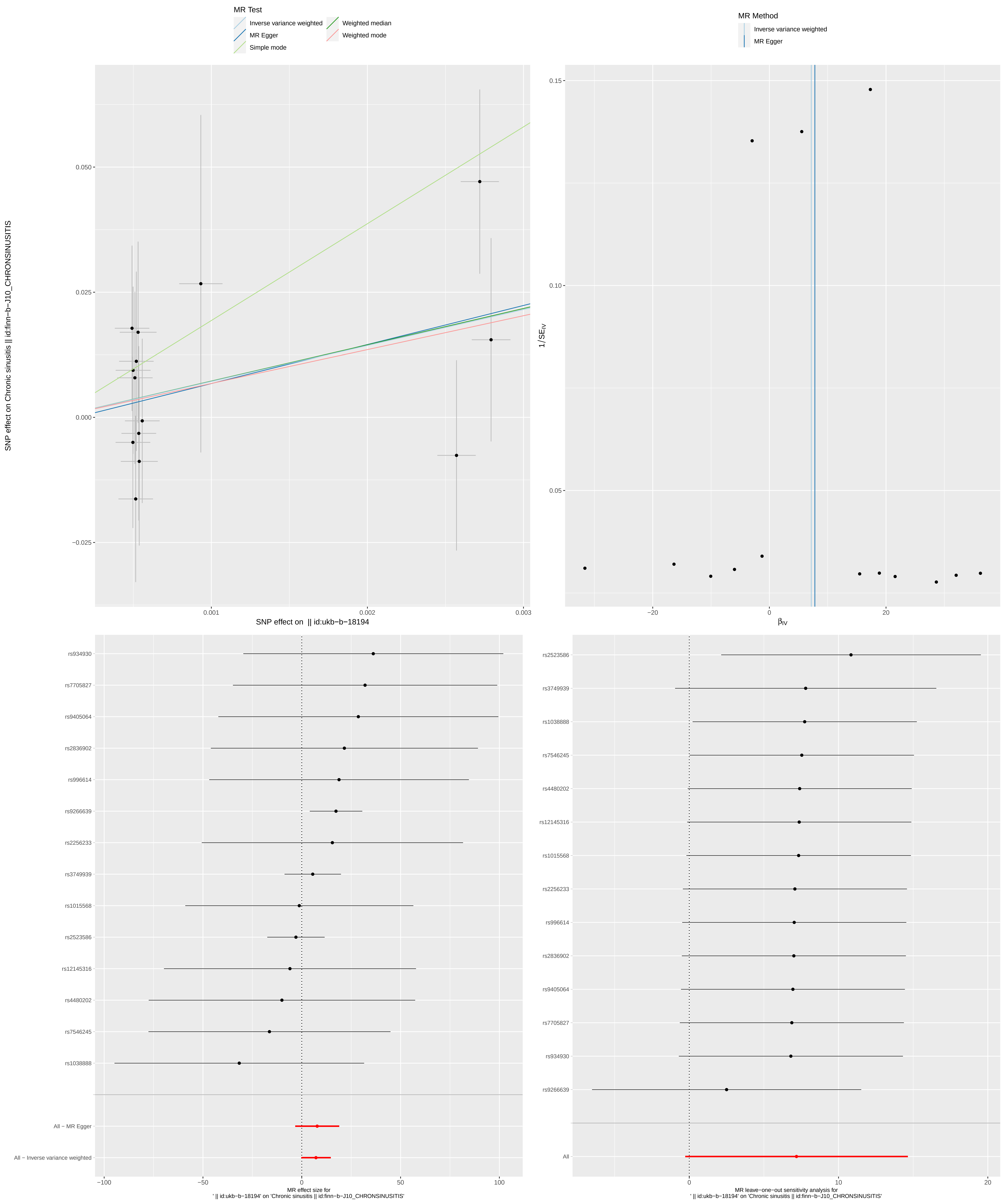
MR Test
Inverse variance weighted
MR Egger
Simple mode
Weighted median
Weighted mode
SNP effect on Chronic sinusitis || id:finn−b−J10_CHRONSINUSITIS
SNP effect on || id:ukb−b−18194
0.050
0.025
0.000
−0.025
0.001
0.002
0.003
MR Method
Inverse variance weighted
MR Egger
1/SE_IV
β_IV
0.15
0.10
0.05
−20
0
20
rs934930
rs7705827
rs9405064
rs2836902
rs996614
rs9266639
rs2256233
rs3749939
rs1015568
rs2523586
rs12145316
rs4480202
rs7546245
rs1038888
All − MR Egger
All − Inverse variance weighted
−100
−50
0
50
100
MR effect size for
' || id:ukb−b−18194' on 'Chronic sinusitis || id:finn−b−J10_CHRONSINUSITIS'
rs2523586
rs3749939
rs1038888
rs7546245
rs4480202
rs12145316
rs1015568
rs2256233
rs996614
rs2836902
rs9405064
rs7705827
rs934930
rs9266639
All
0
10
20
MR leave−one−out sensitivity analysis for
' || id:ukb−b−18194' on 'Chronic sinusitis || id:finn−b−J10_CHRONSINUSITIS'

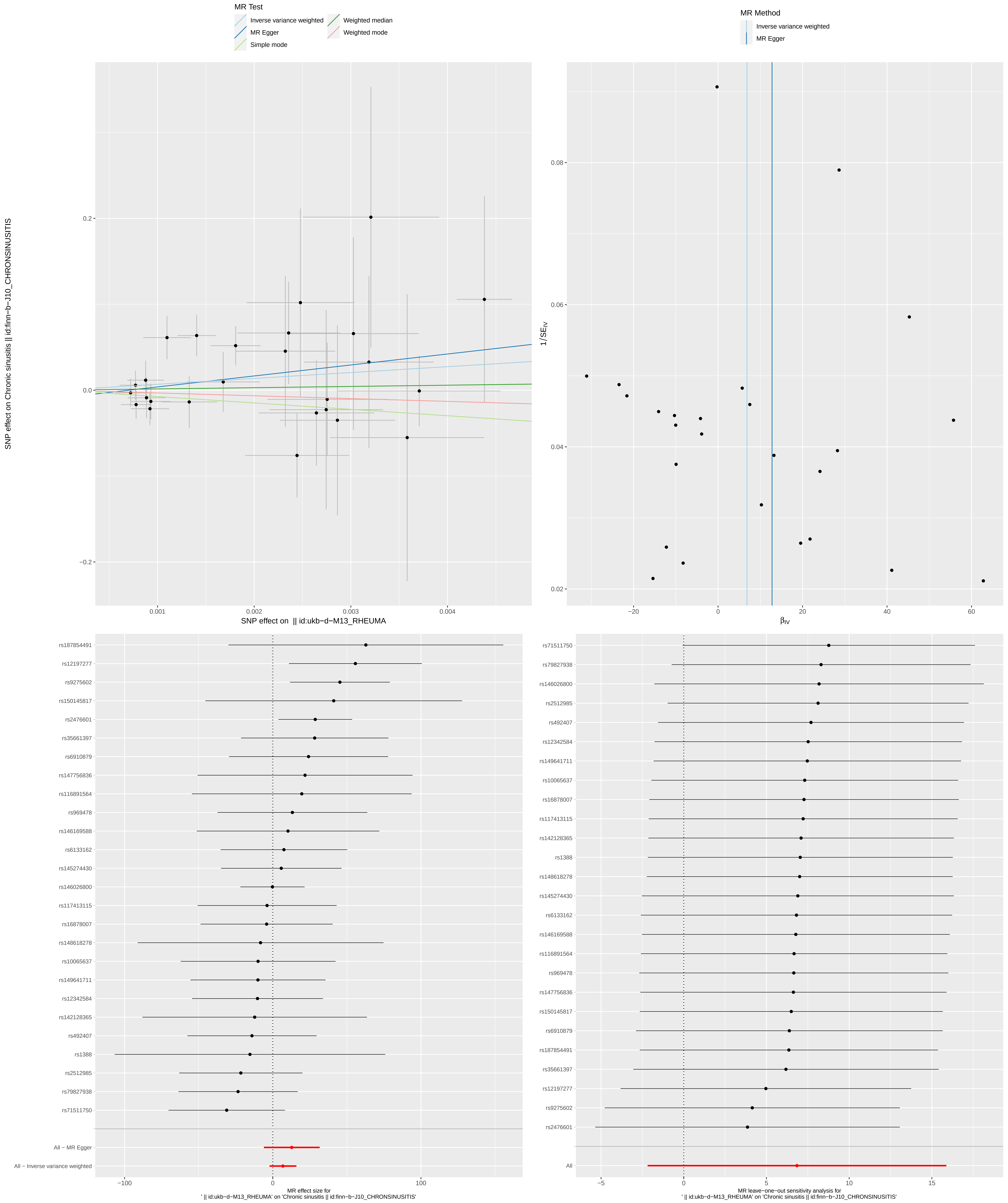

MR Test
Inverse variance weighted
MR Egger
Simple mode
Weighted median
Weighted mode
SNP effect on Chronic sinusitis || id:finn-b-J10_CHRONSINUSITIS
SNP effect on || id:ukb-d-M13_RHEUMA
0.2
0.0
-0.2
0.001
0.002
0.003
0.004
MR Method
Inverse variance weighted
MR Egger
1/SE_IV
β_IV
0.08
0.06
0.04
0.02
-20
0
20
40
60
rs187854491
rs12197277
rs9275602
rs150145817
rs2476601
rs35661397
rs6910879
rs147756836
rs116891564
rs969478
rs146169588
rs6133162
rs145274430
rs146026800
rs117413115
rs16878007
rs148618278
rs10065637
rs149641711
rs12342584
rs142128365
rs492407
rs1388
rs2512985
rs79827938
rs71511750
All - MR Egger
All - Inverse variance weighted
-100
0
100
MR effect size for
' || id:ukb-d-M13_RHEUMA' on 'Chronic sinusitis || id:finn-b-J10_CHRONSINUSITIS'
rs71511750
rs79827938
rs146026800
rs2512985
rs492407
rs12342584
rs149641711
rs10065637
rs16878007
rs117413115
rs142128365
rs1388
rs148618278
rs145274430
rs6133162
rs146169588
rs116891564
rs969478
rs147756836
rs150145817
rs6910879
rs187854491
rs35661397
rs12197277
rs9275602
rs2476601
All
-5
0
5
10
15
MR leave-one-out sensitivity analysis for
' || id:ukb-d-M13_RHEUMA' on 'Chronic sinusitis || id:finn-b-J10_CHRONSINUSITIS'

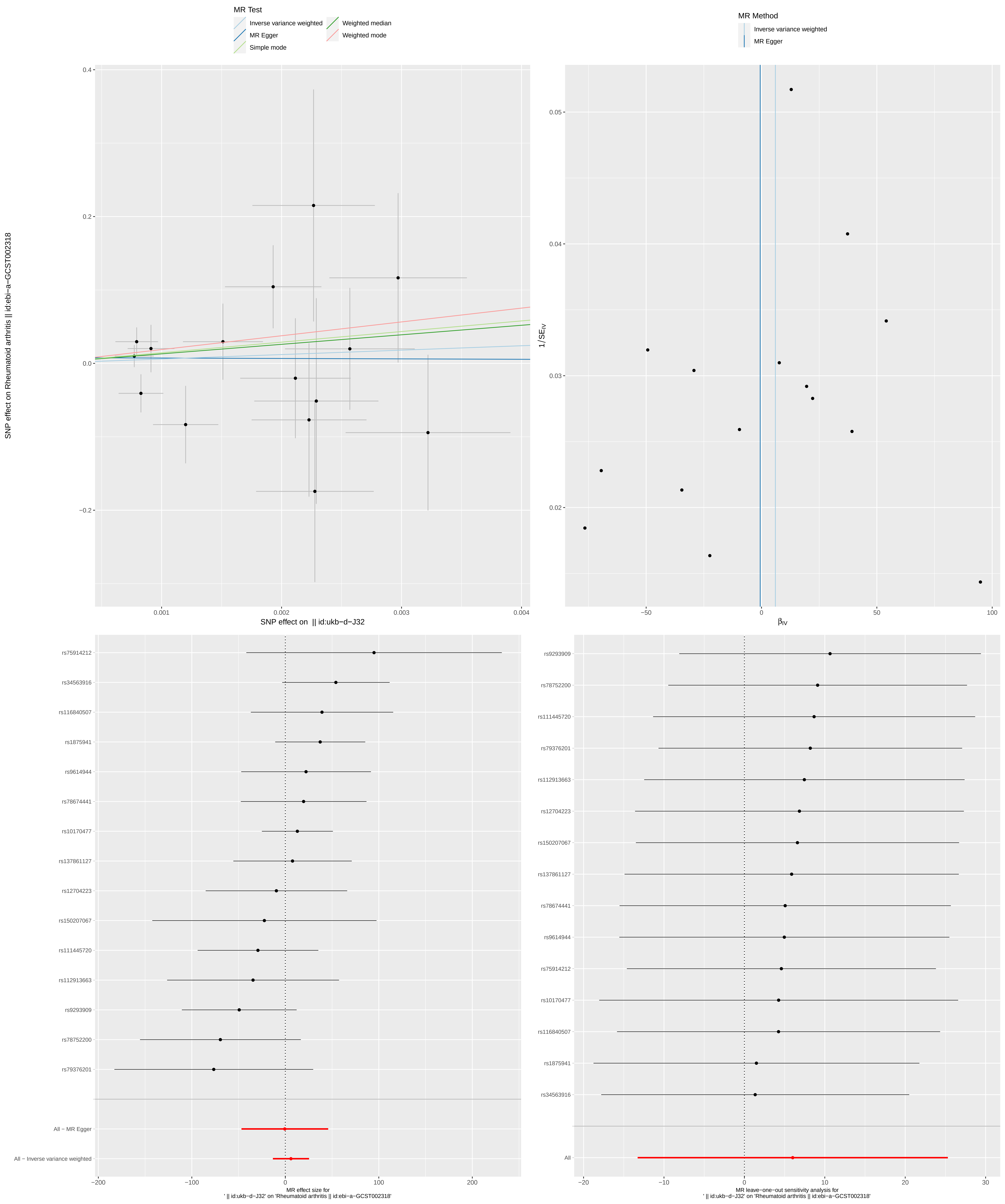
MR Test
Inverse variance weighted
MR Egger
Simple mode
Weighted median
Weighted mode
SNP effect on Rheumatoid arthritis || id:ebi−a−GCST002318
SNP effect on || id:ukb−d−J32
MR Method
Inverse variance weighted
MR Egger
1/SE_IV
β_IV
rs75914212
rs34563916
rs116840507
rs1875941
rs9614944
rs78674441
rs10170477
rs137861127
rs12704223
rs150207067
rs111445720
rs112913663
rs9293909
rs78752200
rs79376201
All − MR Egger
All − Inverse variance weighted
MR effect size for
' || id:ukb−d−J32' on 'Rheumatoid arthritis || id:ebi−a−GCST002318'
rs9293909
rs78752200
rs111445720
rs79376201
rs112913663
rs12704223
rs150207067
rs137861127
rs78674441
rs9614944
rs75914212
rs10170477
rs116840507
rs1875941
rs34563916
All
MR leave−one−out sensitivity analysis for
' || id:ukb−d−J32' on 'Rheumatoid arthritis || id:ebi−a−GCST002318'

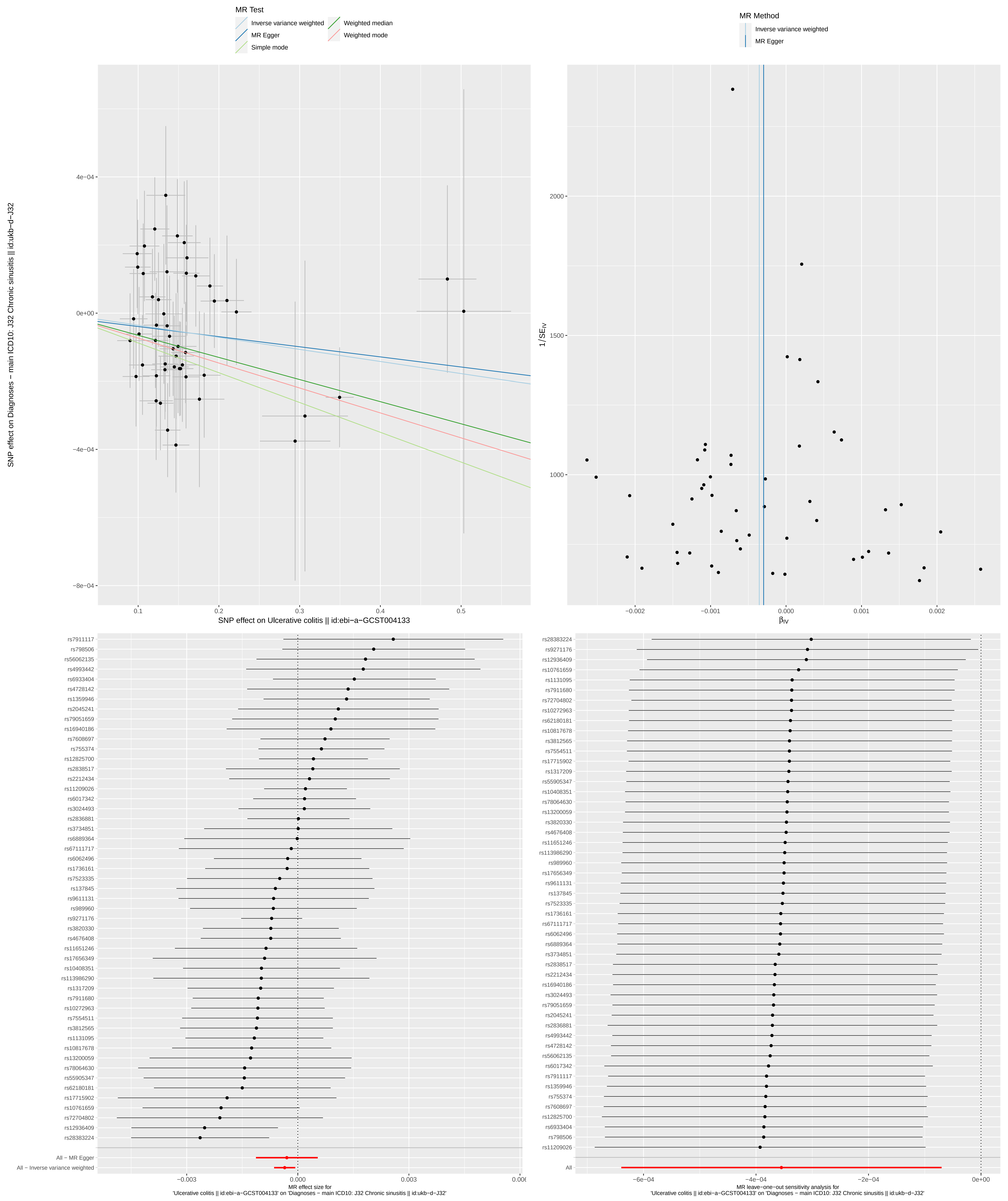
MR Test
Inverse variance weighted
MR Egger
Simple mode
Weighted median
Weighted mode
SNP effect on Diagnoses − main ICD10: J32 Chronic sinusitis || id:ukb−d−J32
SNP effect on Ulcerative colitis || id:ebi−a−GCST004133
4e−04
0e+00
−4e−04
−8e−04
0.1
0.2
0.3
0.4
0.5
MR Method
Inverse variance weighted
MR Egger
1/SE_IV
β_IV
2000
1500
1000
−0.002
−0.001
0.000
0.001
0.002
rs7911117
rs798506
rs56062135
rs4993442
rs6933404
rs4728142
rs1359946
rs2045241
rs79051659
rs16940186
rs7608697
rs755374
rs12825700
rs2838517
rs2212434
rs11209026
rs6017342
rs3024493
rs2836881
rs3734851
rs6889364
rs67111717
rs6062496
rs1736161
rs7523335
rs137845
rs9611131
rs989960
rs9271176
rs3820330
rs4676408
rs11651246
rs17656349
rs10408351
rs113986290
rs1317209
rs7911680
rs10272963
rs7554511
rs3812565
rs1131095
rs10817678
rs13200059
rs78064630
rs55905347
rs62180181
rs17715902
rs10761659
rs72704802
rs12936409
rs28383224
All − MR Egger
All − Inverse variance weighted
−0.003
0.000
0.003
0.006
MR effect size for
'Ulcerative colitis || id:ebi−a−GCST004133' on 'Diagnoses − main ICD10: J32 Chronic sinusitis || id:ukb−d−J32'
rs28383224
rs9271176
rs12936409
rs10761659
rs1131095
rs7911680
rs72704802
rs10272963
rs62180181
rs10817678
rs3812565
rs7554511
rs17715902
rs1317209
rs55905347
rs10408351
rs78064630
rs13200059
rs3820330
rs4676408
rs11651246
rs113986290
rs989960
rs17656349
rs9611131
rs137845
rs7523335
rs1736161
rs67111717
rs6062496
rs6889364
rs3734851
rs2838517
rs2212434
rs16940186
rs3024493
rs79051659
rs2045241
rs2836881
rs4993442
rs4728142
rs56062135
rs6017342
rs7911117
rs1359946
rs755374
rs7608697
rs12825700
rs6933404
rs798506
rs11209026
All
−6e−04
−4e−04
−2e−04
0e+00
MR leave−one−out sensitivity analysis for
'Ulcerative colitis || id:ebi−a−GCST004133' on 'Diagnoses − main ICD10: J32 Chronic sinusitis || id:ukb−d−J32'

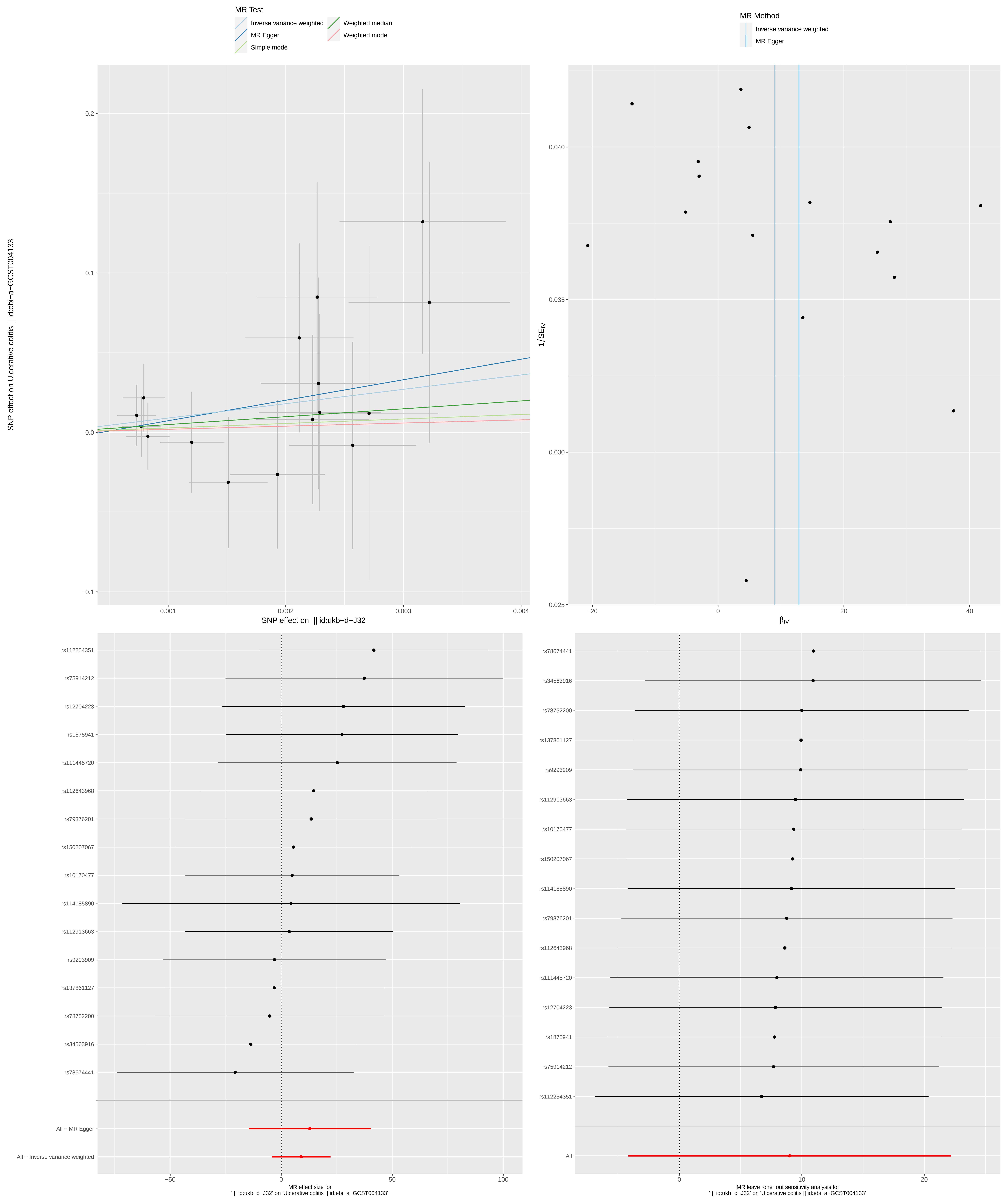

MR Test
Inverse variance weighted
MR Egger
Simple mode
Weighted median
Weighted mode
SNP effect on Ulcerative colitis || id:ebi−a−GCST004133
SNP effect on || id:ukb−d−J32
MR Method
Inverse variance weighted
MR Egger
1/SE_IV
β_IV
rs112254351
rs75914212
rs12704223
rs1875941
rs111445720
rs112643968
rs79376201
rs150207067
rs10170477
rs114185890
rs112913663
rs9293909
rs137861127
rs78752200
rs34563916
rs78674441
All − MR Egger
All − Inverse variance weighted
MR effect size for
' || id:ukb−d−J32' on 'Ulcerative colitis || id:ebi−a−GCST004133'
All
MR leave−one−out sensitivity analysis for
' || id:ukb−d−J32' on 'Ulcerative colitis || id:ebi−a−GCST004133'

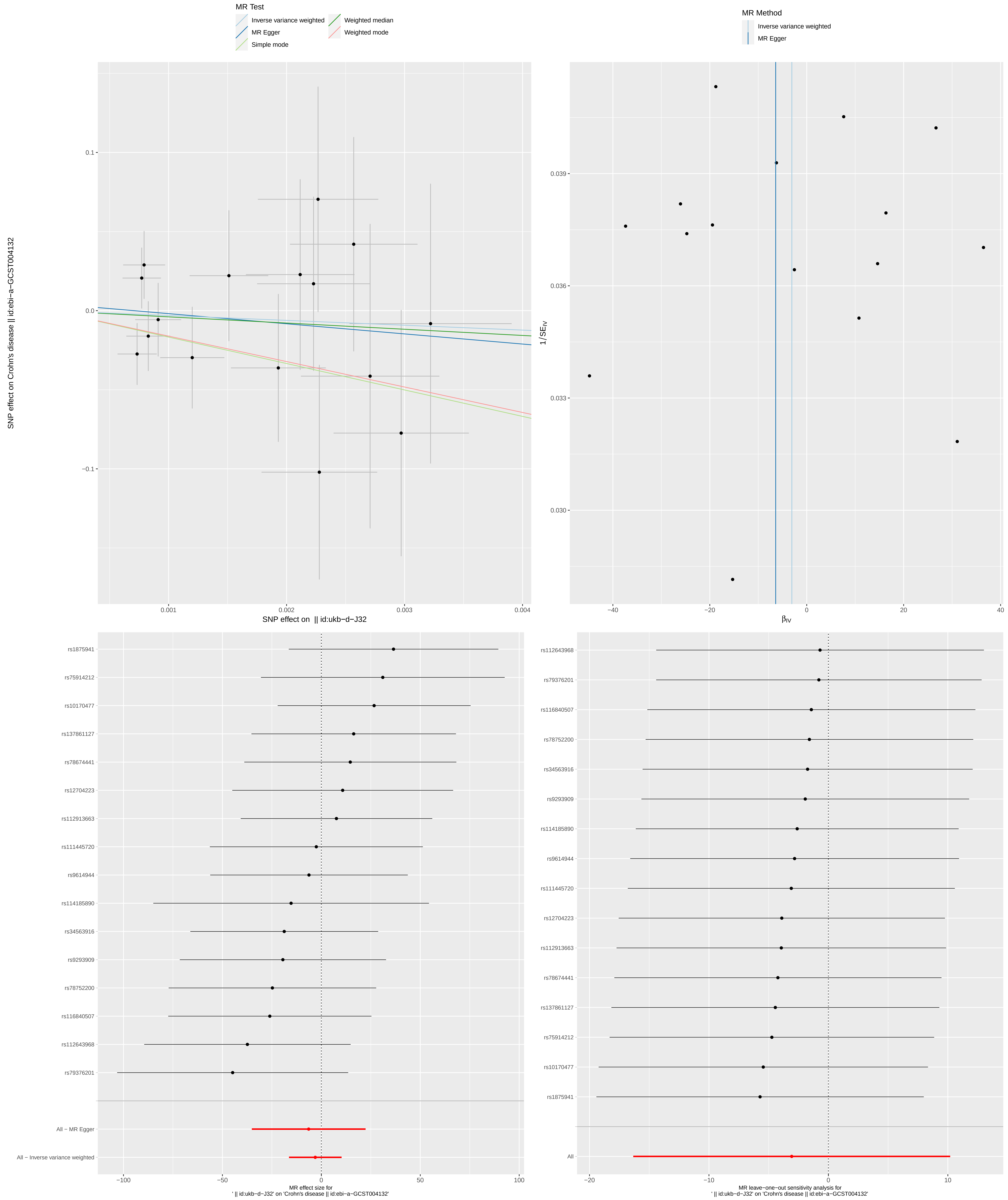
MR Test
Inverse variance weighted
MR Egger
Simple mode
Weighted median
Weighted mode
SNP effect on Crohn's disease || id:ebi−a−GCST004132
SNP effect on || id:ukb−d−J32
MR Method
Inverse variance weighted
MR Egger
1/SE_IV
β_IV
rs1875941
rs75914212
rs10170477
rs137861127
rs78674441
rs12704223
rs112913663
rs111445720
rs9614944
rs114185890
rs34563916
rs9293909
rs78752200
rs116840507
rs112643968
rs79376201
All − MR Egger
All − Inverse variance weighted
MR effect size for
' || id:ukb−d−J32' on 'Crohn's disease || id:ebi−a−GCST004132'
All
MR leave−one−out sensitivity analysis for
' || id:ukb−d−J32' on 'Crohn's disease || id:ebi−a−GCST004132'

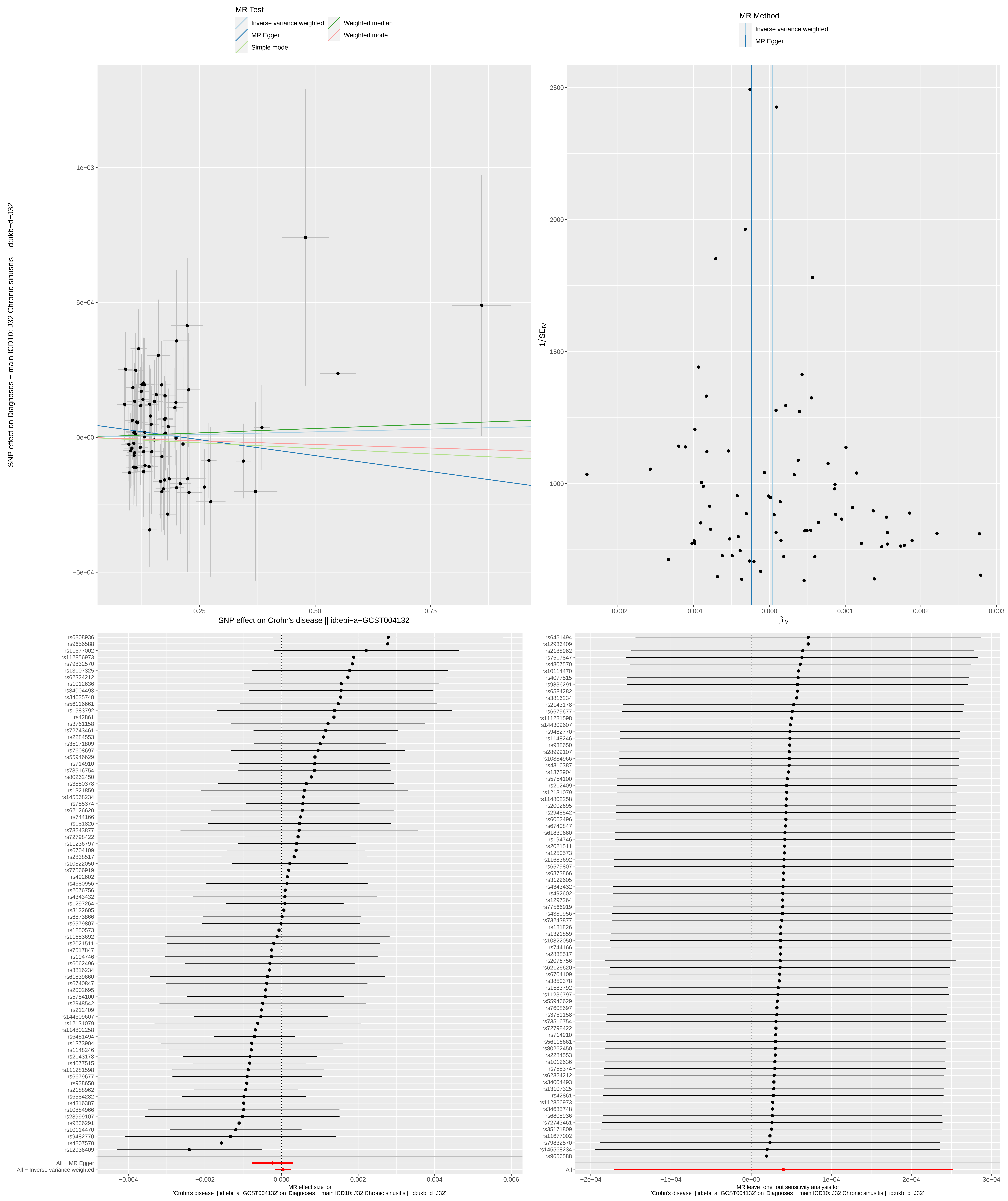

MR Test
Inverse variance weighted
MR Egger
Simple mode
Weighted median
Weighted mode
SNP effect on Diagnoses − main ICD10: J32 Chronic sinusitis || id:ukb−d−J32
SNP effect on Crohn's disease || id:ebi−a−GCST004132
1e−03
5e−04
0e+00
−5e−04
0.25
0.50
0.75
MR Method
Inverse variance weighted
MR Egger
1/SE_IV
β_IV
2500
2000
1500
1000
−0.002
−0.001
0.000
0.001
0.002
0.003
All − MR Egger
All − Inverse variance weighted
MR effect size for
'Crohn's disease || id:ebi−a−GCST004132' on 'Diagnoses − main ICD10: J32 Chronic sinusitis || id:ukb−d−J32'
−0.004
−0.002
0.000
0.002
0.004
0.006
All
MR leave−one−out sensitivity analysis for
'Crohn's disease || id:ebi−a−GCST004132' on 'Diagnoses − main ICD10: J32 Chronic sinusitis || id:ukb−d−J32'
−2e−04
−1e−04
0e+00
1e−04
2e−04
3e−04

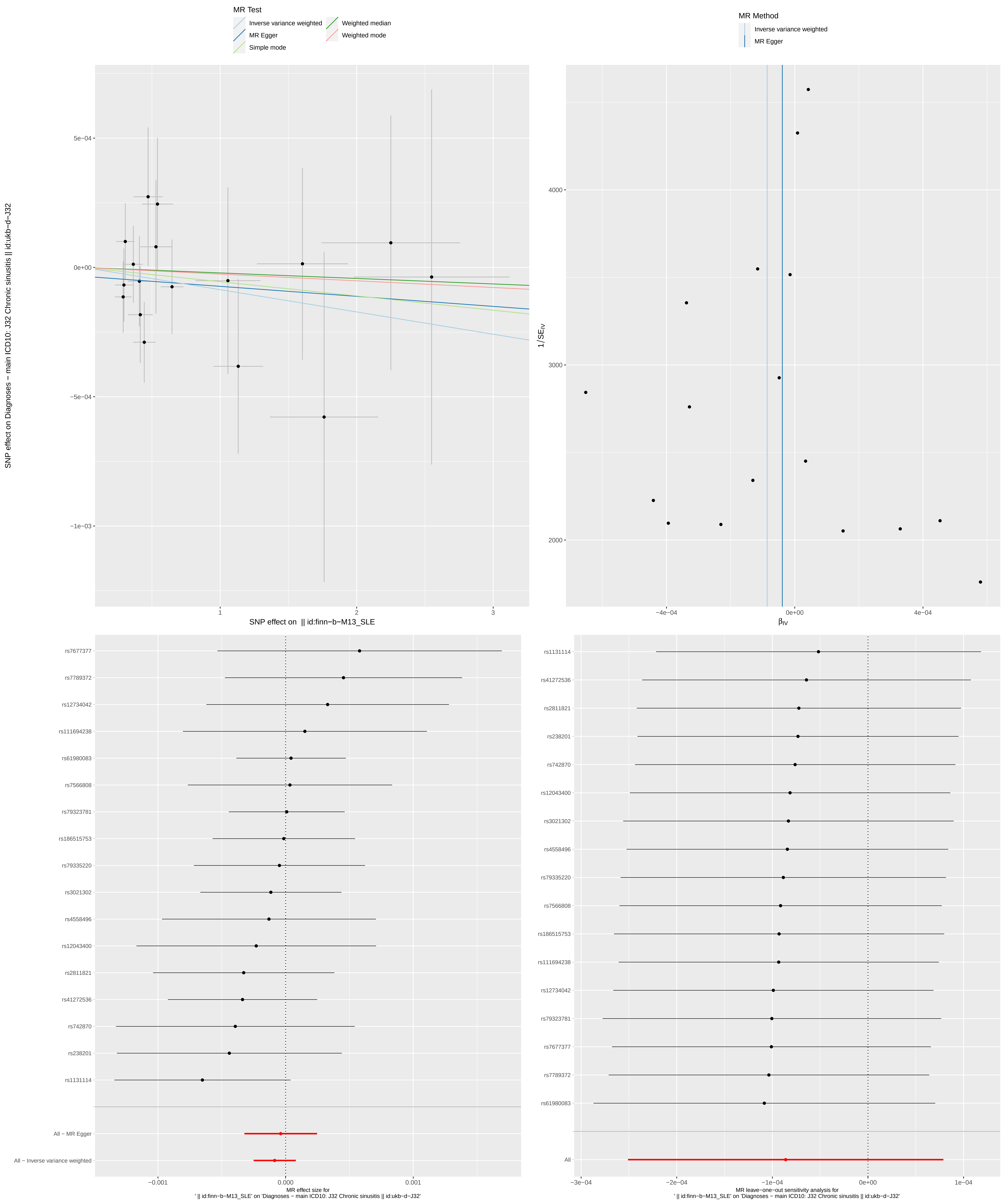

MR Test
Inverse variance weighted
MR Egger
Simple mode
Weighted median
Weighted mode
SNP effect on Diagnoses − main ICD10: J32 Chronic sinusitis || id:ukb−d−J32
5e−04
0e+00
−5e−04
−1e−03
1
2
3
SNP effect on || id:finn−b−M13_SLE
MR Method
Inverse variance weighted
MR Egger
$1/SE_{IV}$
4000
3000
2000
−4e−04
0e+00
4e−04
β_{IV}
rs7677377
rs7789372
rs12734042
rs111694238
rs61980083
rs7566808
rs79323781
rs186515753
rs79335220
rs3021302
rs4558496
rs12043400
rs2811821
rs41272536
rs742870
rs238201
rs1131114
All − MR Egger
All − Inverse variance weighted
−0.001
0.000
0.001
MR effect size for
' || id:finn−b−M13_SLE' on 'Diagnoses − main ICD10: J32 Chronic sinusitis || id:ukb−d−J32'
rs1131114
rs41272536
rs2811821
rs238201
rs742870
rs12043400
rs3021302
rs4558496
rs79335220
rs7566808
rs186515753
rs111694238
rs12734042
rs79323781
rs7677377
rs7789372
rs61980083
All
−3e−04
−2e−04
−1e−04
0e+00
1e−04
MR leave−one−out sensitivity analysis for
' || id:finn−b−M13_SLE' on 'Diagnoses − main ICD10: J32 Chronic sinusitis || id:ukb−d−J32'

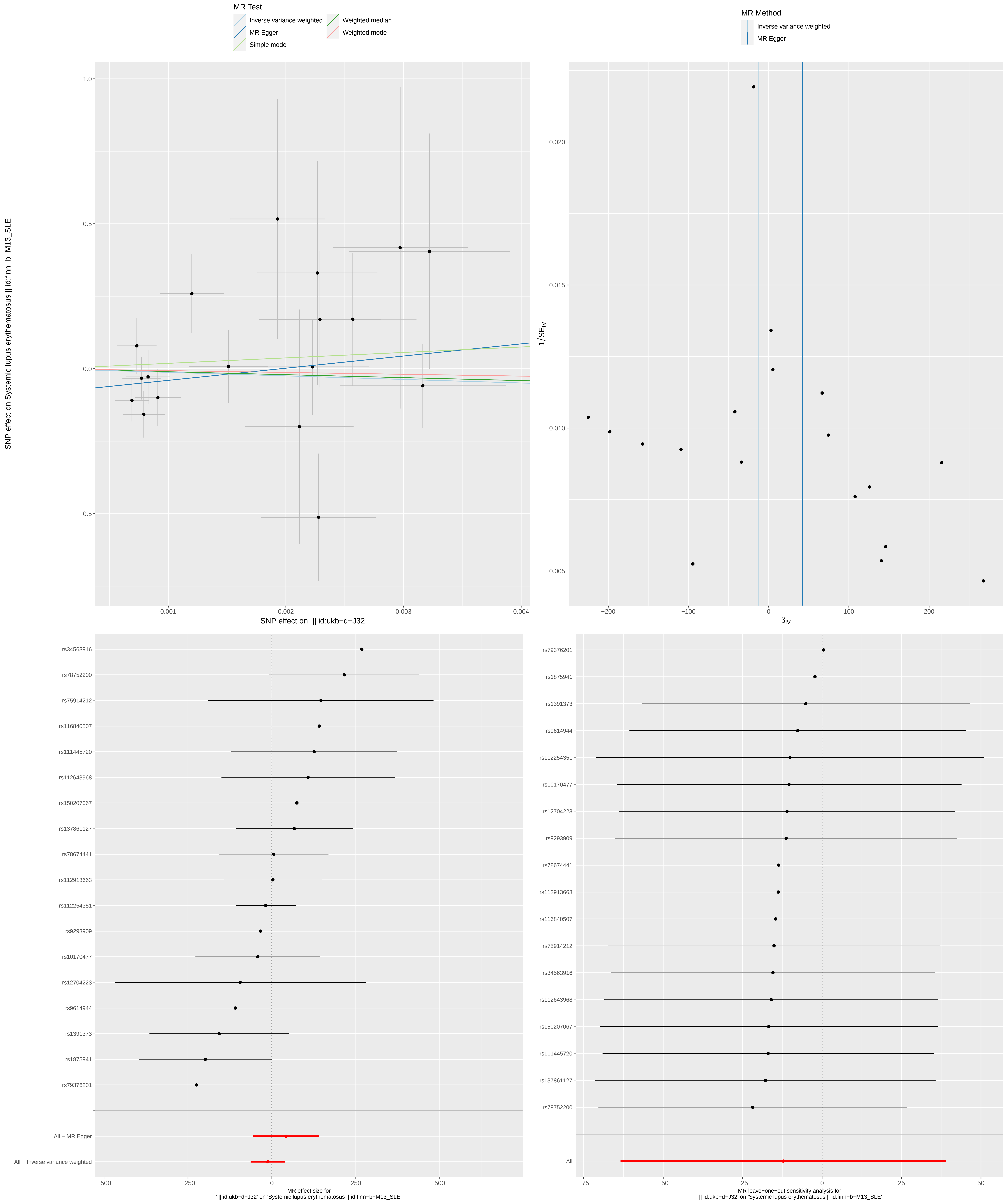
MR Test
Inverse variance weighted
MR Egger
Simple mode
Weighted median
Weighted mode
SNP effect on Systemic lupus erythematosus || id:finn−b−M13_SLE
SNP effect on || id:ukb−d−J32
1.0
0.5
0.0
−0.5
0.001
0.002
0.003
0.004
MR Method
Inverse variance weighted
MR Egger
$1/SE_{IV}$
β_{IV}
0.020
0.015
0.010
0.005
−200
−100
0
100
200
rs34563916
rs78752200
rs75914212
rs116840507
rs111445720
rs112643968
rs150207067
rs137861127
rs78674441
rs112913663
rs112254351
rs9293909
rs10170477
rs12704223
rs9614944
rs1391373
rs1875941
rs79376201
All − MR Egger
All − Inverse variance weighted
−500
−250
0
250
500
MR effect size for
' || id:ukb−d−J32' on 'Systemic lupus erythematosus || id:finn−b−M13_SLE'
rs79376201
rs1875941
rs1391373
rs9614944
rs112254351
rs10170477
rs12704223
rs9293909
rs78674441
rs112913663
rs116840507
rs75914212
rs34563916
rs112643968
rs150207067
rs111445720
rs137861127
rs78752200
All
−75
−50
−25
0
25
50
MR leave−one−out sensitivity analysis for
' || id:ukb−d−J32' on 'Systemic lupus erythematosus || id:finn−b−M13_SLE'

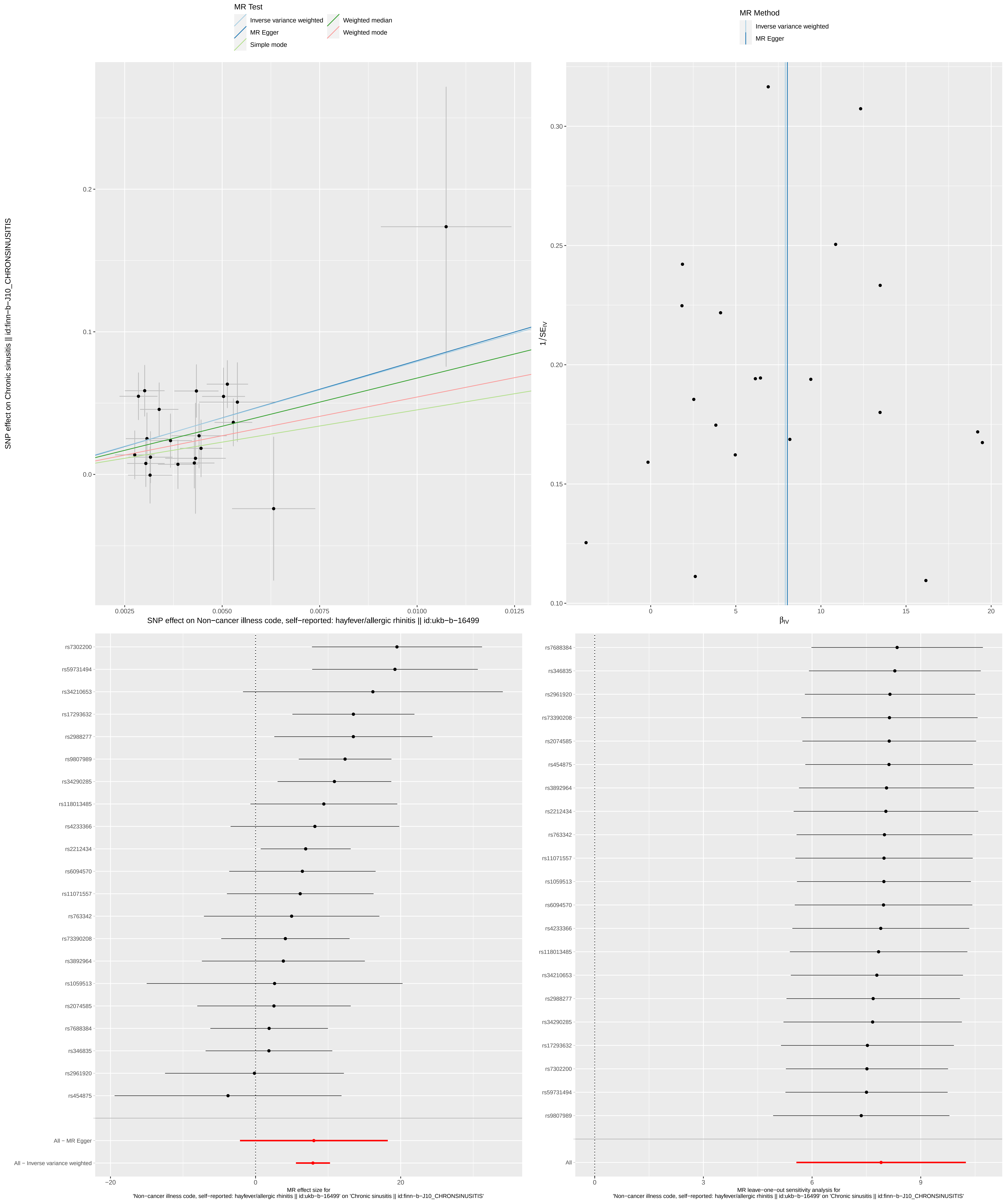
MR Test
Inverse variance weighted
MR Egger
Simple mode
Weighted median
Weighted mode
MR Method
Inverse variance weighted
MR Egger
SNP effect on Chronic sinusitis || id:finn−b−J10_CHRONSINUSITIS
SNP effect on Non−cancer illness code, self−reported: hayfever/allergic rhinitis || id:ukb−b−16499
1/SE_IV
β_IV
rs7302200
rs59731494
rs34210653
rs17293632
rs2988277
rs9807989
rs34290285
rs118013485
rs4233366
rs2212434
rs6094570
rs11071557
rs763342
rs73390208
rs3892964
rs1059513
rs2074585
rs7688384
rs346835
rs2961920
rs454875
All − MR Egger
All − Inverse variance weighted
MR effect size for
'Non−cancer illness code, self−reported: hayfever/allergic rhinitis || id:ukb−b−16499' on 'Chronic sinusitis || id:finn−b−J10_CHRONSINUSITIS'
rs7688384
rs346835
rs2961920
rs73390208
rs2074585
rs454875
rs3892964
rs2212434
rs763342
rs11071557
rs1059513
rs6094570
rs4233366
rs118013485
rs34210653
rs2988277
rs34290285
rs17293632
rs7302200
rs59731494
rs9807989
All
MR leave−one−out sensitivity analysis for
'Non−cancer illness code, self−reported: hayfever/allergic rhinitis || id:ukb−b−16499' on 'Chronic sinusitis || id:finn−b−J10_CHRONSINUSITIS'

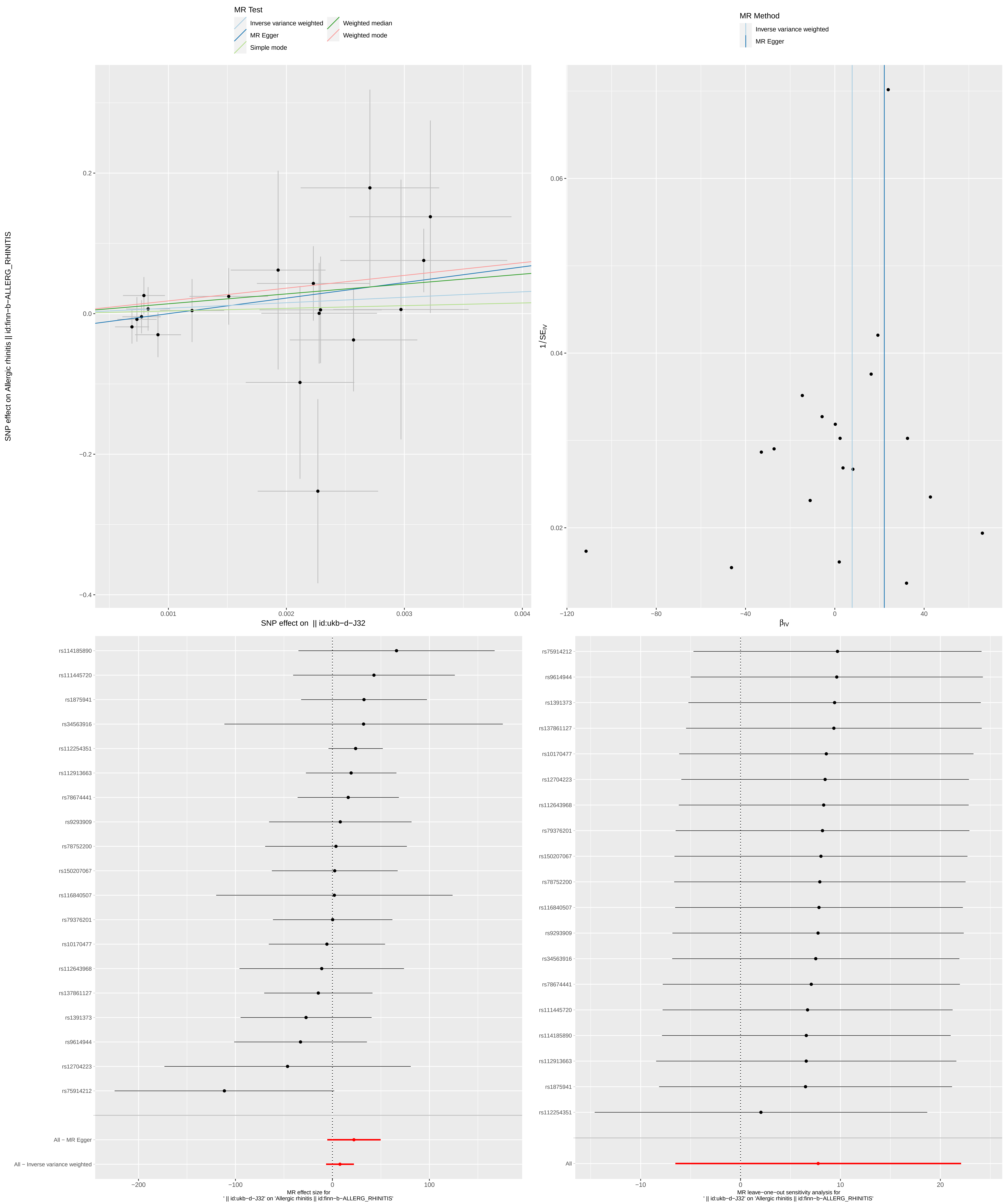
MR Test
Inverse variance weighted
MR Egger
Simple mode
Weighted median
Weighted mode
SNP effect on Allergic rhinitis || id:finn−b−ALLERG_RHINITIS
SNP effect on || id:ukb−d−J32
MR Method
Inverse variance weighted
MR Egger
1/SE_IV
β_IV
rs114185890
rs111445720
rs1875941
rs34563916
rs112254351
rs112913663
rs78674441
rs9293909
rs78752200
rs150207067
rs116840507
rs79376201
rs10170477
rs112643968
rs137861127
rs1391373
rs9614944
rs12704223
rs75914212
All − MR Egger
All − Inverse variance weighted
MR effect size for
' || id:ukb−d−J32' on 'Allergic rhinitis || id:finn−b−ALLERG_RHINITIS'
rs75914212
rs9614944
rs1391373
rs137861127
rs10170477
rs12704223
rs112643968
rs79376201
rs150207067
rs78752200
rs116840507
rs9293909
rs34563916
rs78674441
rs111445720
rs114185890
rs112913663
rs1875941
rs112254351
All
MR leave−one−out sensitivity analysis for
' || id:ukb−d−J32' on 'Allergic rhinitis || id:finn−b−ALLERG_RHINITIS'

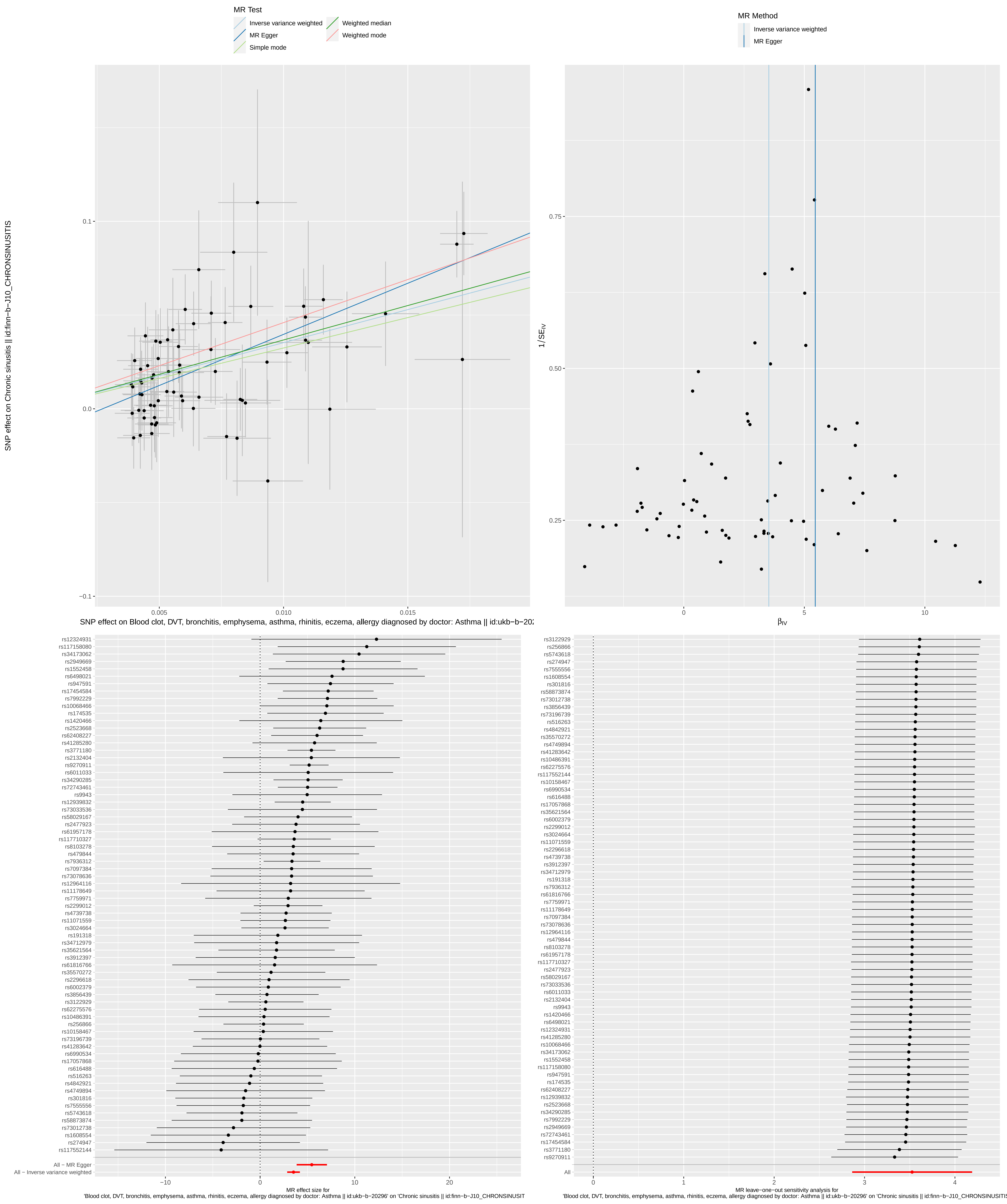
MR Test
Inverse variance weighted
MR Egger
Simple mode
Weighted median
Weighted mode
MR Method
Inverse variance weighted
MR Egger
SNP effect on Chronic sinusitis || id:finn−b−J10_CHRONSINUSITIS
SNP effect on Blood clot, DVT, bronchitis, emphysema, asthma, rhinitis, eczema, allergy diagnosed by doctor: Asthma || id:ukb−b−202
0.1
0.0
−0.1
0.005
0.010
0.015
1/SE_IV
0.75
0.50
0.25
β_IV
0
5
10
rs12324931
rs117158080
rs34173062
rs2949669
rs1552458
rs6498021
rs947591
rs17454584
rs7992229
rs10068466
rs174535
rs1420466
rs2523668
rs62408227
rs41285280
rs3771180
rs2132404
rs9270911
rs6011033
rs34290285
rs72743461
rs9943
rs12939832
rs73033536
rs58029167
rs2477923
rs61957178
rs117710327
rs8103278
rs479844
rs7936312
rs7097384
rs73078636
rs12964116
rs11178649
rs7759971
rs2299012
rs4739738
rs11071559
rs3024664
rs191318
rs34712979
rs35621564
rs3912397
rs61816766
rs35570272
rs2296618
rs6002379
rs3856439
rs3122929
rs62275576
rs10486391
rs256866
rs10158467
rs73196739
rs41283642
rs6990534
rs17057868
rs616488
rs516263
rs4842921
rs4749894
rs301816
rs7555556
rs5743618
rs58873874
rs73012738
rs1608554
rs274947
rs117552144
All − MR Egger
All − Inverse variance weighted
−10
0
10
20
MR effect size for
'Blood clot, DVT, bronchitis, emphysema, asthma, rhinitis, eczema, allergy diagnosed by doctor: Asthma || id:ukb−b−20296' on 'Chronic sinusitis || id:finn−b−J10_CHRONSINUSIT
rs3122929
rs256866
rs5743618
rs274947
rs7555556
rs1608554
rs301816
rs58873874
rs73012738
rs3856439
rs73196739
rs516263
rs4842921
rs35570272
rs4749894
rs41283642
rs10486391
rs62275576
rs117552144
rs10158467
rs6990534
rs616488
rs17057868
rs35621564
rs6002379
rs2299012
rs3024664
rs11071559
rs2296618
rs4739738
rs3912397
rs34712979
rs191318
rs7936312
rs61816766
rs7759971
rs11178649
rs7097384
rs73078636
rs12964116
rs479844
rs8103278
rs61957178
rs117710327
rs2477923
rs58029167
rs73033536
rs6011033
rs2132404
rs9943
rs1420466
rs6498021
rs12324931
rs41285280
rs10068466
rs34173062
rs1552458
rs117158080
rs947591
rs174535
rs62408227
rs12939832
rs2523668
rs34290285
rs7992229
rs2949669
rs72743461
rs17454584
rs3771180
rs9270911
All
0
1
2
3
4
MR leave−one−out sensitivity analysis for
'Blood clot, DVT, bronchitis, emphysema, asthma, rhinitis, eczema, allergy diagnosed by doctor: Asthma || id:ukb−b−20296' on 'Chronic sinusitis || id:finn−b−J10_CHRONSINUSITIS

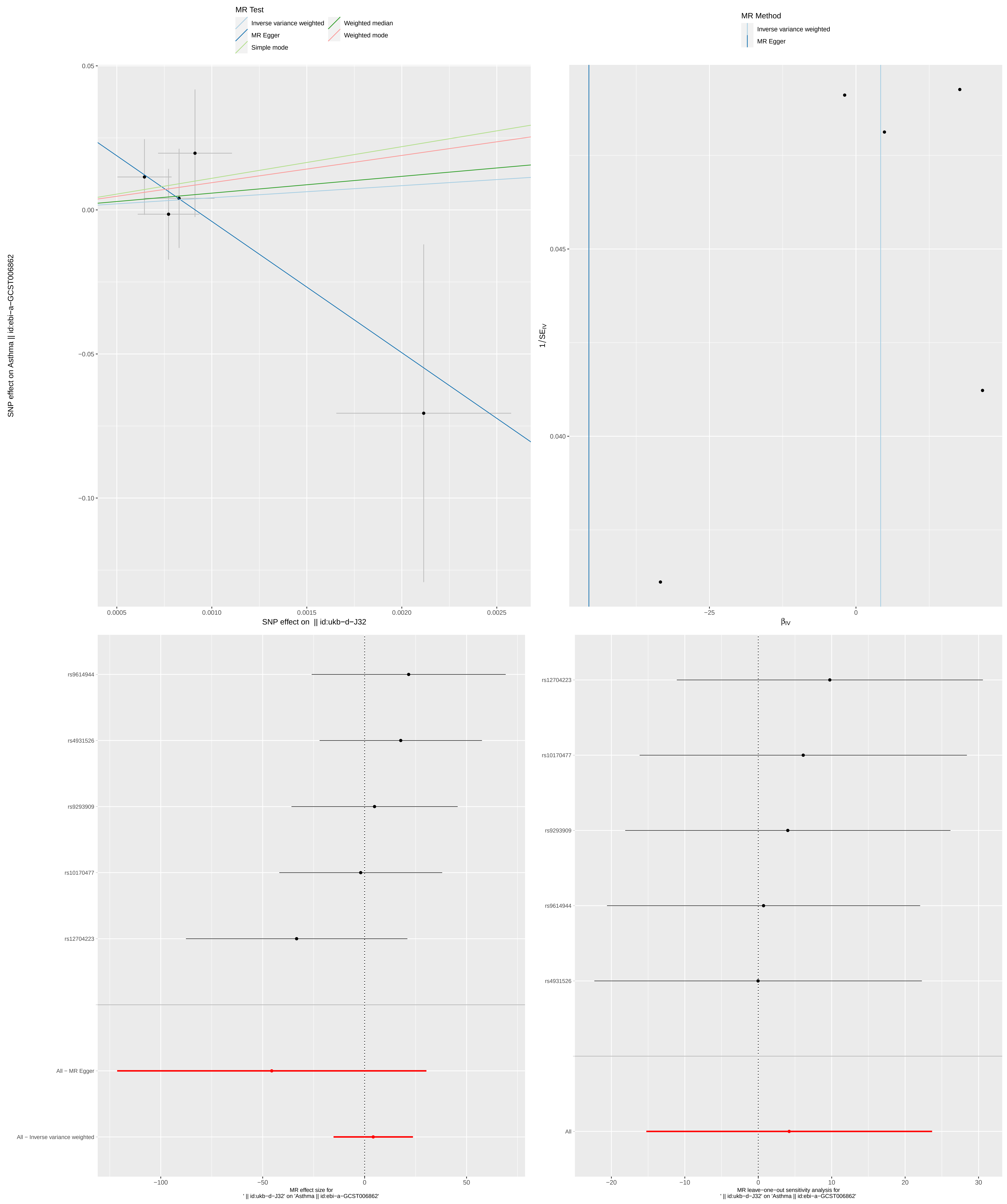
MR Test
Inverse variance weighted
MR Egger
Simple mode
Weighted median
Weighted mode
SNP effect on Asthma || id:ebi−a−GCST006862
SNP effect on || id:ukb−d−J32
0.05
0.00
−0.05
−0.10
0.0005
0.0010
0.0015
0.0020
0.0025
MR Method
Inverse variance weighted
MR Egger
1/SE$_{IV}$
β_{IV}
0.045
0.040
−25
0
rs9614944
rs4931526
rs9293909
rs10170477
rs12704223
All − MR Egger
All − Inverse variance weighted
−100
−50
0
50
MR effect size for
' || id:ukb−d−J32' on 'Asthma || id:ebi−a−GCST006862'
rs12704223
rs10170477
rs9293909
rs9614944
rs4931526
All
−20
−10
0
10
20
30
MR leave−one−out sensitivity analysis for
' || id:ukb−d−J32' on 'Asthma || id:ebi−a−GCST006862'

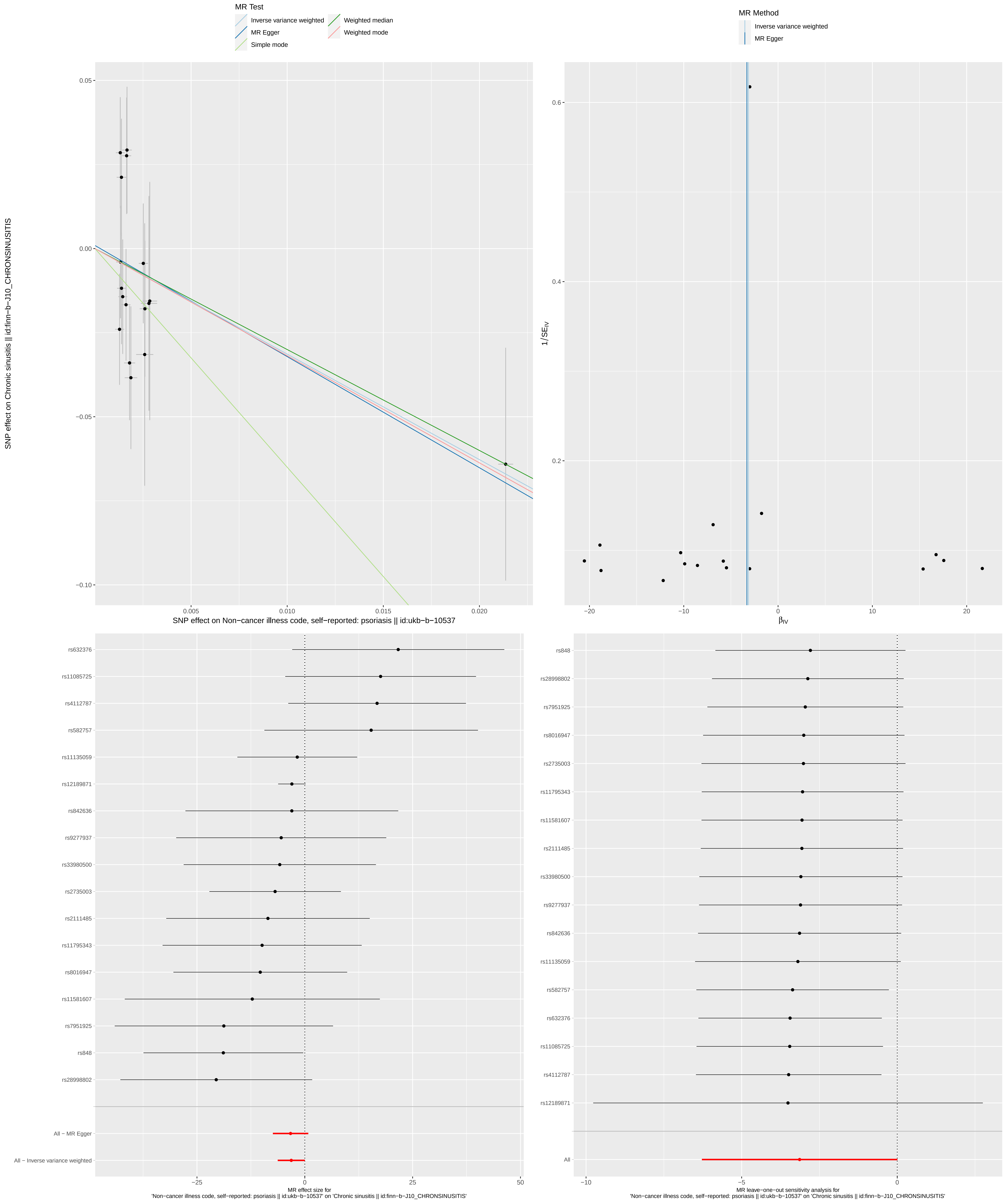

MR Test
Inverse variance weighted
MR Egger
Simple mode
Weighted median
Weighted mode
SNP effect on Chronic sinusitis || id:finn−b−J10_CHRONSINUSITIS
SNP effect on Non−cancer illness code, self−reported: psoriasis || id:ukb−b−10537
0.05
0.00
−0.05
−0.10
0.005
0.010
0.015
0.020
MR Method
Inverse variance weighted
MR Egger
1/SE_IV
β_IV
0.6
0.4
0.2
−20
−10
0
10
20
rs632376
rs11085725
rs4112787
rs582757
rs11135059
rs12189871
rs842636
rs9277937
rs33980500
rs2735003
rs2111485
rs11795343
rs8016947
rs11581607
rs7951925
rs848
rs28998802
All − MR Egger
All − Inverse variance weighted
−25
0
25
50
MR effect size for
'Non−cancer illness code, self−reported: psoriasis || id:ukb−b−10537' on 'Chronic sinusitis || id:finn−b−J10_CHRONSINUSITIS'
rs848
rs28998802
rs7951925
rs8016947
rs2735003
rs11795343
rs11581607
rs2111485
rs33980500
rs9277937
rs842636
rs11135059
rs582757
rs632376
rs11085725
rs4112787
rs12189871
All
−10
−5
0
MR leave−one−out sensitivity analysis for
'Non−cancer illness code, self−reported: psoriasis || id:ukb−b−10537' on 'Chronic sinusitis || id:finn−b−J10_CHRONSINUSITIS'

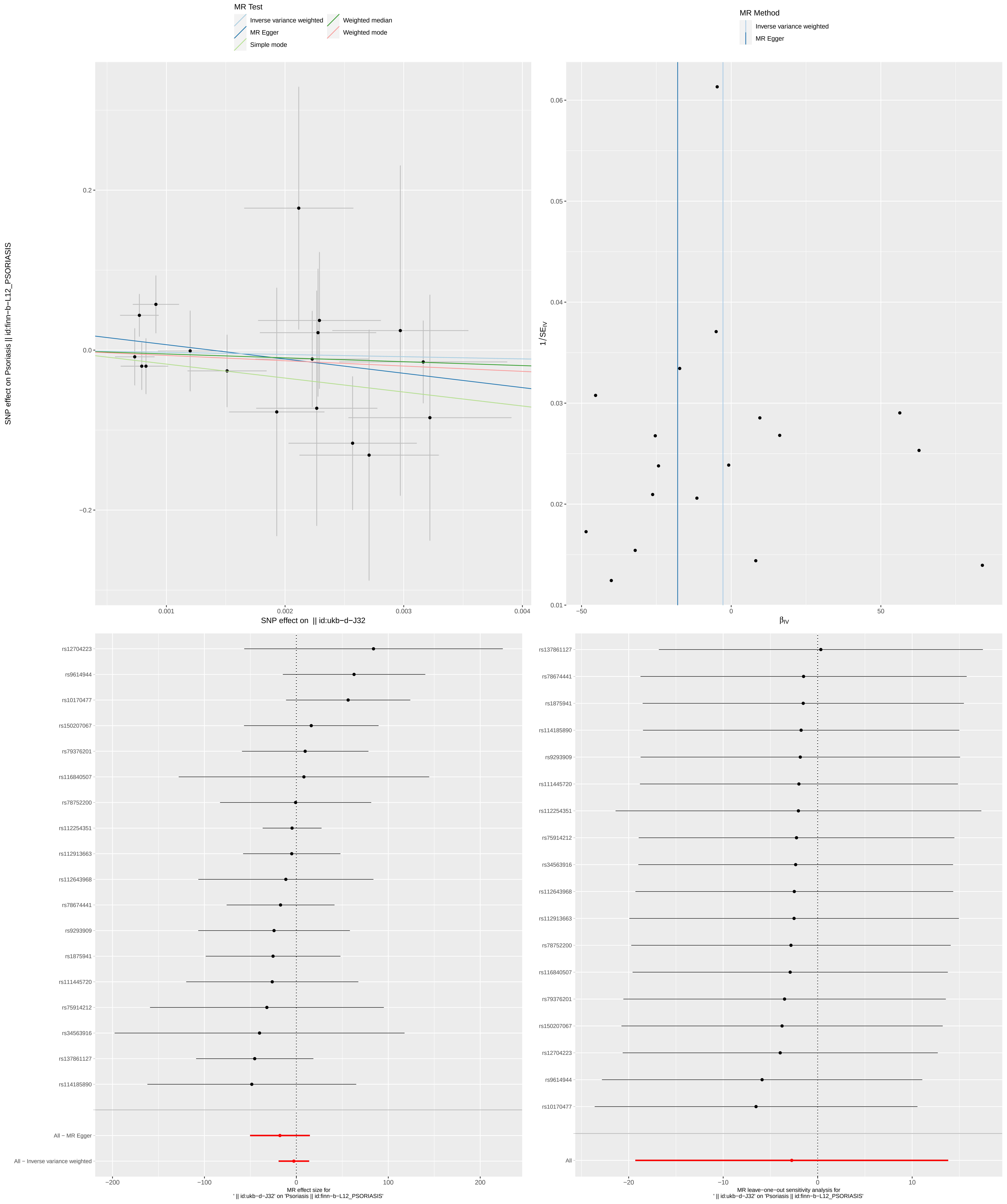
MR Test
Inverse variance weighted
MR Egger
Simple mode
Weighted median
Weighted mode
SNP effect on Psoriasis || id:finn−b−L12_PSORIASIS
SNP effect on || id:ukb−d−J32
MR Method
Inverse variance weighted
MR Egger
1/SE_IV
β_IV
rs12704223
rs9614944
rs10170477
rs150207067
rs79376201
rs116840507
rs78752200
rs112254351
rs112913663
rs112643968
rs78674441
rs9293909
rs1875941
rs111445720
rs75914212
rs34563916
rs137861127
rs114185890
All − MR Egger
All − Inverse variance weighted
MR effect size for
' || id:ukb−d−J32' on 'Psoriasis || id:finn−b−L12_PSORIASIS'
rs137861127
rs78674441
rs1875941
rs114185890
rs9293909
rs111445720
rs112254351
rs75914212
rs34563916
rs112643968
rs112913663
rs78752200
rs116840507
rs79376201
rs150207067
rs12704223
rs9614944
rs10170477
All
MR leave−one−out sensitivity analysis for
' || id:ukb−d−J32' on 'Psoriasis || id:finn−b−L12_PSORIASIS'

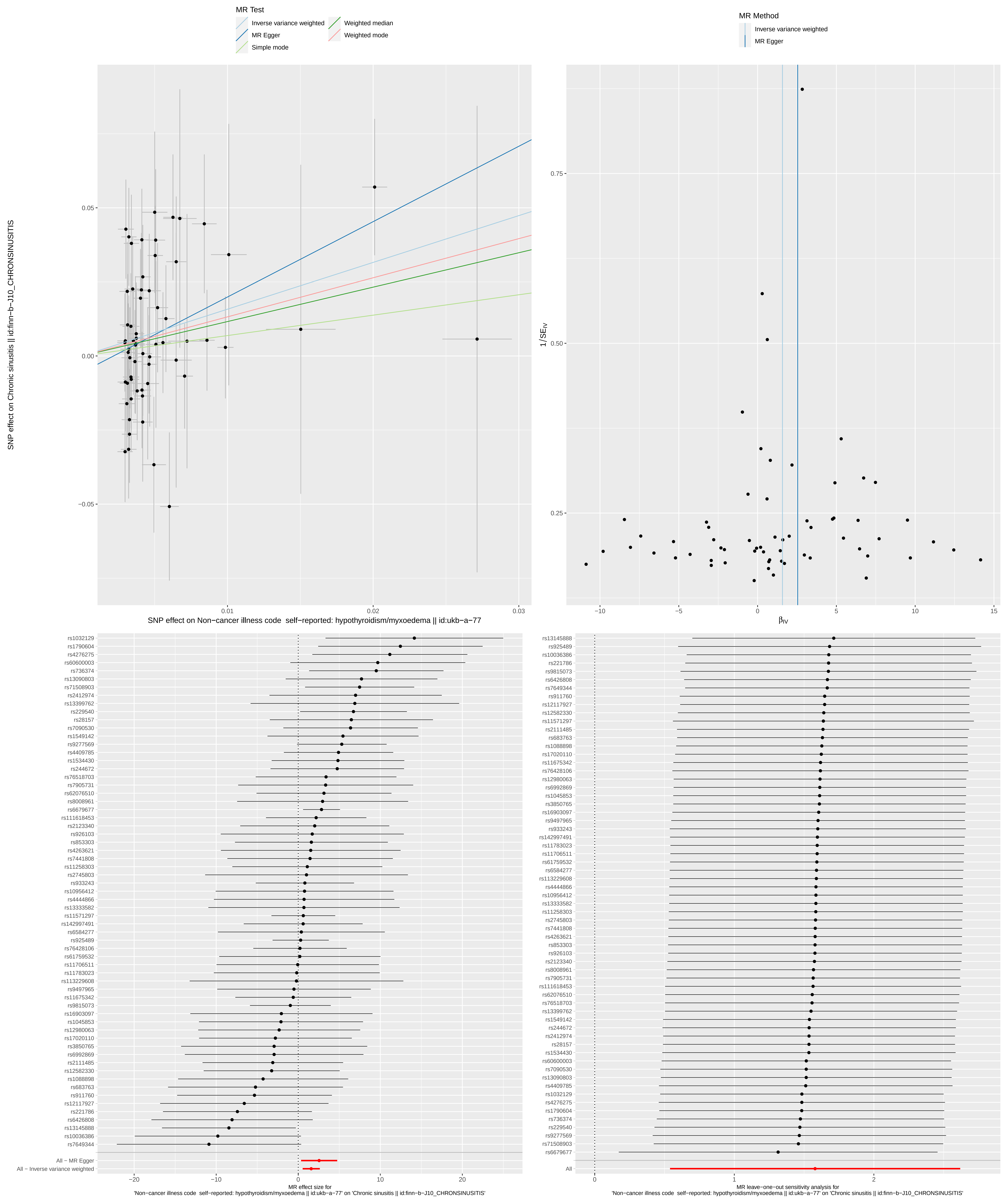

MR Test
Inverse variance weighted
MR Egger
Simple mode
Weighted median
Weighted mode
SNP effect on Chronic sinusitis || id:finn−b−J10_CHRONSINUSITIS
SNP effect on Non−cancer illness code self−reported: hypothyroidism/myxoedema || id:ukb−a−77
0.05
0.00
−0.05
0.01
0.02
0.03
MR Method
Inverse variance weighted
MR Egger
1/SE_IV
β_IV
0.75
0.50
0.25
−10
−5
0
5
10
15
rs1032129
rs1790604
rs4276275
rs60600003
rs736374
rs13090803
rs71508903
rs2412974
rs13399762
rs229540
rs28157
rs7090530
rs1549142
rs9277569
rs4409785
rs1534430
rs244672
rs76518703
rs7905731
rs62076510
rs8008961
rs6679677
rs111618453
rs2123340
rs926103
rs853303
rs4263621
rs7441808
rs11258303
rs2745803
rs933243
rs10956412
rs4444866
rs13333582
rs11571297
rs142997491
rs6584277
rs925489
rs76428106
rs61759532
rs11706511
rs11783023
rs113229608
rs9497965
rs11675342
rs9815073
rs16903097
rs1045853
rs12980063
rs17020110
rs3850765
rs6992869
rs2111485
rs12582330
rs1088898
rs683763
rs911760
rs12117927
rs221786
rs6426808
rs13145888
rs10036386
rs7649344
All − MR Egger
All − Inverse variance weighted
−20
−10
0
10
20
MR effect size for
'Non−cancer illness code self−reported: hypothyroidism/myxoedema || id:ukb−a−77' on 'Chronic sinusitis || id:finn−b−J10_CHRONSINUSITIS'
rs13145888
rs925489
rs10036386
rs221786
rs9815073
rs6426808
rs7649344
rs911760
rs12117927
rs12582330
rs11571297
rs2111485
rs683763
rs1088898
rs17020110
rs11675342
rs76428106
rs12980063
rs6992869
rs1045853
rs3850765
rs16903097
rs9497965
rs933243
rs142997491
rs11783023
rs11706511
rs61759532
rs6584277
rs113229608
rs4444866
rs10956412
rs13333582
rs11258303
rs2745803
rs7441808
rs4263621
rs853303
rs926103
rs2123340
rs8008961
rs7905731
rs111618453
rs62076510
rs76518703
rs13399762
rs1549142
rs244672
rs2412974
rs28157
rs1534430
rs60600003
rs7090530
rs13090803
rs4409785
rs1032129
rs4276275
rs1790604
rs736374
rs229540
rs9277569
rs71508903
rs6679677
All
0
1
2
MR leave−one−out sensitivity analysis for
'Non−cancer illness code self−reported: hypothyroidism/myxoedema || id:ukb−a−77' on 'Chronic sinusitis || id:finn−b−J10_CHRONSINUSITIS'

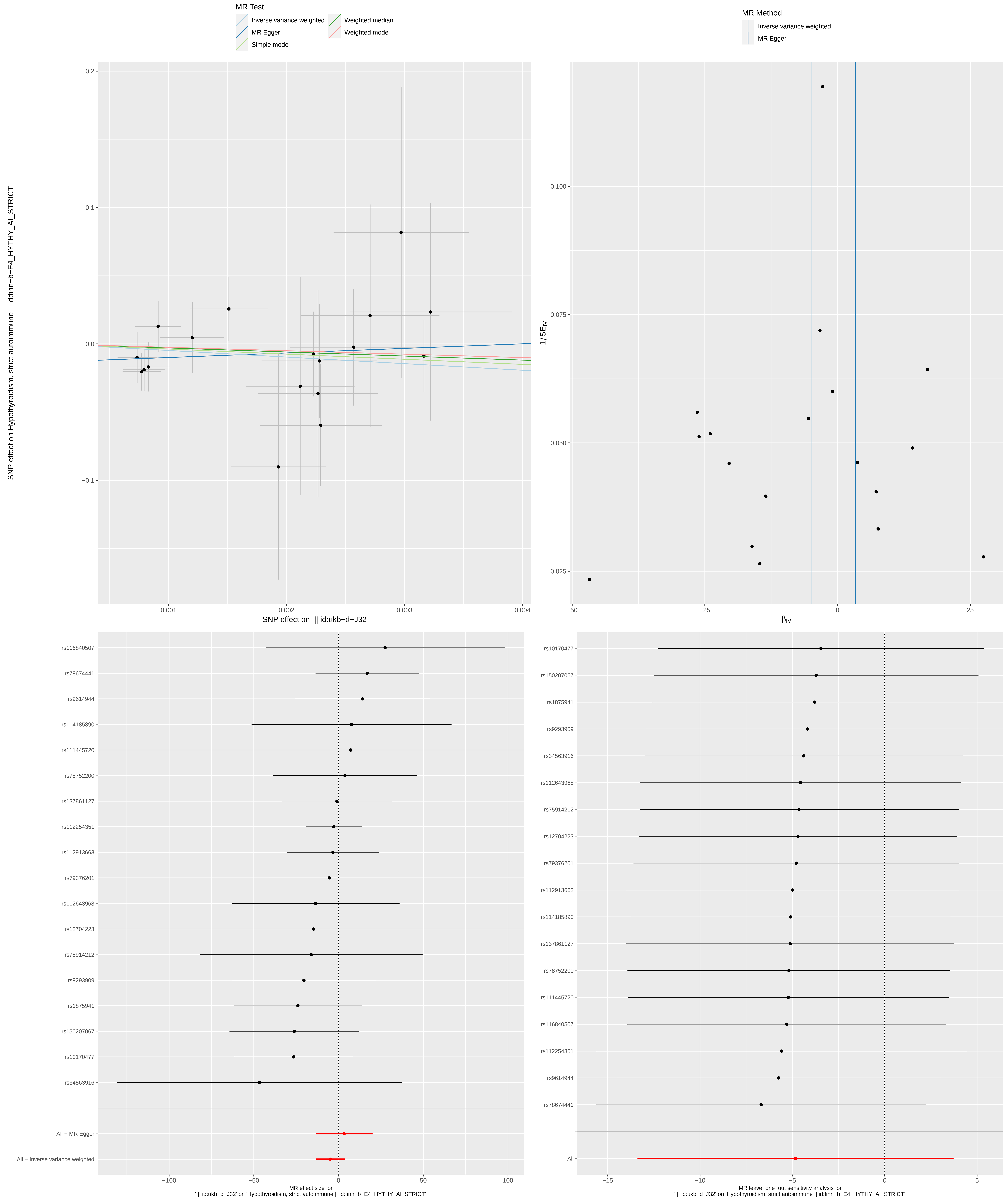

MR Test
Inverse variance weighted
MR Egger
Simple mode
Weighted median
Weighted mode
SNP effect on Hypothyroidism, strict autoimmune || id:finn-b-E4_HYTHY_AI_STRICT
0.2
0.1
0.0
−0.1
0.001
0.002
0.003
0.004
SNP effect on || id:ukb-d-J32
MR Method
Inverse variance weighted
MR Egger
1/SE_IV
0.100
0.075
0.050
0.025
−50
−25
0
25
β_IV
rs116840507
rs78674441
rs9614944
rs114185890
rs111445720
rs78752200
rs137861127
rs112254351
rs112913663
rs79376201
rs112643968
rs12704223
rs75914212
rs9293909
rs1875941
rs150207067
rs10170477
rs34563916
All − MR Egger
All − Inverse variance weighted
−100
−50
0
50
100
MR effect size for
' || id:ukb-d-J32' on 'Hypothyroidism, strict autoimmune || id:finn-b-E4_HYTHY_AI_STRICT'
rs10170477
rs150207067
rs1875941
rs9293909
rs34563916
rs112643968
rs75914212
rs12704223
rs79376201
rs112913663
rs114185890
rs137861127
rs78752200
rs111445720
rs116840507
rs112254351
rs9614944
rs78674441
All
−15
−10
−5
0
5
MR leave−one−out sensitivity analysis for
' || id:ukb-d-J32' on 'Hypothyroidism, strict autoimmune || id:finn-b-E4_HYTHY_AI_STRICT'
