# Supplementary material for: Genetic correlation between chronic sinusitis and autoimmune diseases
Source: Front Allergy. 2024 Sep 24;5:1387774. doi: 10.3389/falgy.2024.1387774 (PMC11458559; doi:10.3389/falgy.2024.1387774)
Supplement: Supplementary file 2 [file Datasheet2.pdf]

exposure	outcome	method	Threshold	nsnp	b	pval	or	or_lci95	or_uci95
TYPE 1	CRS	mr_MaxLik	######	26	0.04793	0.002722	1.049	1.0167	1.0825
TYPE 1	CRS	mr_Median	######	26	0.0673	0.003932	1.07	1.0218	1.11968
TYPE 1	CRS	mr_mbe	######	26	0.07765	0.001225	1.081	1.031	1.13283
CRS	TYPE 1	mr_MaxLik	######	16	9.31707	0.523375	11126	######	3E+16
CRS	TYPE 1	mr_Median	######	16	21.5957	0.264752	2E+09	######	7.28E+25
CRS	TYPE 1	mr_mbe	######	16	21.3799	0.448966	2E+09	######	2.10E+33
AS	CRS	mr_MaxLik	######	14	7.27079	0.056731	1438	0.8119	2545970
AS	CRS	mr_Median	######	14	7.42471	0.177484	1677	0.0345	8.1E+07
AS	CRS	mr_mbe	######	14	6.76935	0.178579	870.7	0.0453	1.7E+07
CRS	AS	mr_MaxLik	######	18	24.4213	0.097464	4E+10	0.0116	1.41E+23
CRS	AS	mr_Median	######	18	0.11544	0.995346	1.122	######	7.9E+16
CRS	AS	mr_mbe	######	18	-5.8034	0.806254	0.003	######	4.2E+17
RA	CRS	mr_MaxLik	######	52	0.00028	0.043799	1	1	1.00055
RA	CRS	mr_Median	######	52	0.00034	0.151975	1	0.9999	1.00081
RA	CRS	mr_mbe	######	52	0.0004	0.071683	1	1	1.00084
CRS	RA	mr_MaxLik	######	4	31.6828	0.046165	6E+13	1.7132	1.9E+27
CRS	RA	mr_Median	######	4	35.3634	0.079625	2E+15	0.0153	3.4E+32
CRS	RA	mr_mbe	######	4	39.0596	0.098022	9E+16	0.0007	1.1E+37
UC	CRS	mr_MaxLik	######	51	-0.0004	0.014065	1	0.9994	1.00E+00
UC	CRS	mr_Median	######	51	-0.0007	0.002578	0.999	######	0.99977
UC	CRS	mr_mbe	######	51	-0.0007	0.035884	0.999	######	0.99995
CRS	UC	mr_MaxLik	######	16	9.3459	0.171189	11452	0.0176	7.5E+09
CRS	UC	mr_Median	######	16	5.01027	0.573522	149.9	######	5.7E+09
CRS	UC	mr_mbe	######	16	1.96703	0.895051	7.149	######	3.5E+13
CD	CRS	mr_MaxLik	######	78	4.09E-05	0.705649	1	0.9998	1.00025
CD	CRS	mr_Median	######	78	6.69E-05	0.688455	1	0.9997	1.00039
CD	CRS	mr_mbe	######	78	-5.31E-05	0.836224	1	0.9994	1.00045
CRS	CD	mr_MaxLik	######	16	-3.0977	0.653164	0.045	######	33303.3
CRS	CD	mr_Median	######	16	-4.0962	0.661776	0.017	######	1553926

CRS	CD	mr_mbe	######	16	-16.09	0.346538	#####	######	3.6E+07
SLE	CRS	mr_MaxLik	######	17	-8.67E-05	0.308847	1	0.9997	1.00008
SLE	CRS	mr_Median	######	17	-2.37E-05	0.83408	1	0.9998	1.0002
SLE	CRS	mr_mbe	######	17	-2.59E-05	0.862395	1	0.9997	1.00027
CRS	SLE	mr_MaxLik	######	18	-11.685	0.661889	#####	######	4.7E+17
CRS	SLE	mr_Median	######	18	-11.029	0.749642	#####	######	4.27E+24
CRS	SLE	mr_mbe	######	18	-6.2956	0.86571	0.002	######	9.01E+28
AR	CRS	mr_MaxLik	######	21	8.2634	1.19E-11	3879	356.05	42266.2
AR	CRS	mr_Median	######	21	6.91585	2.86E-05	1008	39.507	25725.1
AR	CRS	mr_mbe	######	21	5.42789	0.051125	227.7	0.9737	53232.2
CRS	AR	mr_MaxLik	######	19	8.11132	0.271054	3332	0.0018	6.2E+09
CRS	AR	mr_Median	######	19	16.3298	0.11332	1E+07	0.0206	7.4E+15
CRS	AR	mr_mbe	######	19	18.1434	0.140183	8E+07	0.0026	2.2E+18
AT	CRS	mr_MaxLik	######	12	4.58E-01	3.26E-22	1.581	1.441	1.73425
AT	CRS	mr_Median	######	12	4.34E-01	4.36E-12	1.544	1.3654	1.74609
AT	CRS	mr_mbe	######	12	4.53E-01	2.15E-08	1.573	1.3425	1.84358
CRS	AT	mr_MaxLik	######	5	4.29786	0.668082	73.54	######	2.5E+10
CRS	AT	mr_Median	######	5	5.96911	0.638355	391.2	######	2.5E+13
CRS	AT	mr_mbe	######	5	9.45386	0.540782	#####	######	1.8E+17
Psoriasis	CRS	mr_MaxLik	######	17	#######	0.056872	0.047	0.002	1.09377
Psoriasis	CRS	mr_Median	######	17	#######	0.053212	0.05	0.0024	1.04249
Psoriasis	CRS	mr_mbe	######	17	#######	0.045052	0.041	0.0018	0.9322
CRS	Psoriasis	mr_MaxLik	######	18	-2.6575	0.756026	#####	######	1338431
CRS	Psoriasis	mr_Median	######	18	-4.8323	0.695713	#####	######	2.62E+08
CRS	Psoriasis	mr_mbe	######	18	-6.6497	0.621303	0.001	######	3.72E+08
Hypothyroidism	CRS	mr_MaxLik	######	63	1.60338	2.69E-03	4.97	1.744	14.162
Hypothyroidism	CRS	mr_Median	######	63	1.32181	8.12E-02	3.75	0.8488	16.5701
Hypothyroidism	CRS	mr_mbe	######	63	1.31974	0.157536	3.742	0.6003	23.3327
CRS	Hypothyroidism	mr_MaxLik	######	18	-4.6949	0.291372	0.009	1E-06	56.0605
CRS	Hypothyroidism	mr_Median	######	18	-2.9862	0.640998	0.05	2E-07	14263.3

CRS	Hypothyroidism	mr_mbe	######	18	-2.5062	0.728935	0.082	6E-08	116862
