# Supplementary material for: Genetic correlation between chronic sinusitis and autoimmune diseases
Source: Front Allergy. 2024 Sep 24;5:1387774. doi: 10.3389/falgy.2024.1387774 (PMC11458559; doi:10.3389/falgy.2024.1387774)
Supplement: Supplementary file 3 [file Datasheet3.pdf]

exposure	outcome	method	pval	pval(BH-corrected	or	or_lci95	or_uci95	FStatistic	
Type 1 diabetes	CRS	MR Egger	0.008	0.04937	1.091	1.02869	1.1578	71.53	
Type 1 diabetes	CRS	Weighted median	0.004	0.02472	1.069	1.02194	1.1185	71.53	
Type 1 diabetes	CRS	Inverse variance weighted	0.003	0.02088	1.048	1.01629	1.0817	71.53	
Type 1 diabetes	CRS	Simple mode	0.045	0.2151	1.101	1.00679	1.2037	71.53	
Type 1 diabetes	CRS	Weighted mode	0.002	0.01546	1.081	1.03495	1.1286	71.53	
CRS	Type 1 diabetes	MR Egger	0.527	0.82949	1E+09	5.00E-19	######	21.41	
CRS	Type 1 diabetes	Weighted median	0.274	0.57167	1E+09	6.43E-08	######	21.41	
CRS	Type 1 diabetes	Inverse variance weighted	0.531	0.82949	8413	4.46E-09	2E+16	21.41	
CRS	Type 1 diabetes	Simple mode	0.491	0.79239	4E+09	9.55E-18	######	21.41	
CRS	Type 1 diabetes	Weighted mode	0.484	0.79239	2E+09	8.70E-17	######	21.41	
AS	CRS	MR Egger	0.194	0.46108	2443	0.03678	2E+08	121.87	
AS	CRS	Weighted median	0.185	0.45028	1409	0.03139	6E+07	121.87	
AS	CRS	Inverse variance weighted	0.059	0.2213	1317	0.76255	2E+06	121.87	
AS	CRS	Simple mode	0.062	0.2213	3E+08	2.17833	3E+16	121.87	
AS	CRS	Weighted mode	0.224	0.50901	870.7	0.02675	3E+07	121.87	
CRS	AS	MR Egger	0.835	0.89296	335.8	1.22E-21	######	21.34	
CRS	AS	Weighted median	0.949	0.95213	0.266	9.28E-19	8E+16	21.34	
CRS	AS	Inverse variance weighted	0.097	0.31423	1E+10	0.0143	######	21.34	
CRS	AS	Simple mode	0.952	0.95213	0.124	8.44E-31	######	21.34	
CRS	AS	Weighted mode	0.804	0.89296	0.003	7.16E-23	1E+17	21.34	
RA	CRS	MR Egger	#####	0.00039	1.125	1.0721	1.1801	128.72	
RA	CRS	Weighted median	#####	1.36E-06	1.13	1.08029	1.1817	128.72	
RA	CRS	Inverse variance weighted	#####	9.55E-10	1.116	1.07995	1.1523	128.72	
RA	CRS	Simple mode	0.268	0.57148	1.057	0.96008	1.1629	128.72	
RA	CRS	Weighted mode	#####	2.45E-05	1.127	1.08215	1.174	128.72	
CRS	RA	MR Egger	0.629	0.86397	2E+10	3.40E-26	######	21.57	
CRS	RA	Weighted median	0.091	0.30233	8E+14	0.00433	######	21.57	
CRS	RA	Inverse variance weighted	0.043	0.2151	4E+13	2.53198	######	21.57	
CRS	RA	Simple mode	0.28	0.57219	7E+15	1.55E-08	######	21.57	

CRS	RA	Weighted mode	0.201	0.46643	9E+16	0.00042	######	21.57	
UC	CRS	MR Egger	0.488	0.79239	1	0.99887	1.0005	70.9	
UC	CRS	Weighted median	0.003	0.02088	0.999	0.99892	0.9998	70.9	
UC	CRS	Inverse variance weighted	0.015	0.0808	1	0.99936	0.9999	70.9	
UC	CRS	Simple mode	0.083	0.28764	0.999	0.99816	1.0001	70.9	
UC	CRS	Weighted mode	0.053	0.22027	0.999	0.99854	1	70.9	
CRS	UC	MR Egger	0.374	0.65596	4E+05	4.65E-07	3E+17	20.95	
CRS	UC	Weighted median	0.569	0.86271	139.5	5.71E-06	3E+09	20.95	
CRS	UC	Inverse variance weighted	0.18	0.45006	8196	0.01557	4E+09	20.95	
CRS	UC	Simple mode	0.855	0.89296	16.64	2.32E-12	1E+14	20.95	
CRS	UC	Weighted mode	0.895	0.91277	7.149	2.74E-12	2E+13	20.95	
CD	CRS	MR Egger	0.389	0.67154	1	0.99923	1.0003	81.43	
CD	CRS	Weighted median	0.693	0.86397	1	0.99974	1.0004	81.43	
CD	CRS	Inverse variance weighted	0.708	0.86397	1	0.99983	1.0003	81.43	
CD	CRS	Simple mode	0.819	0.89296	1	0.99921	1.0006	81.43	
CD	CRS	Weighted mode	0.837	0.89296	1	0.99944	1.0005	81.43	
CRS	CD	MR Egger	0.669	0.86397	0.002	5.58E-16	5E+09	21.53	
CRS	CD	Weighted median	0.671	0.86397	0.02	2.80E-10	1E+06	21.53	
CRS	CD	Inverse variance weighted	0.65	0.86397	0.046	8.05E-08	26592	21.53	
CRS	CD	Simple mode	0.346	0.64027	#####	1.36E-22	2E+07	21.53	
CRS	CD	Weighted mode	0.357	0.64927	#####	3.95E-22	3E+07	21.53	
SLE	CRS	MR Egger	0.793	0.89296	1	0.99968	1.0002	25.45	
SLE	CRS	Weighted median	0.854	0.89296	1	0.99975	1.0002	25.45	
SLE	CRS	Inverse variance weighted	0.306	0.58877	1	0.99975	1.0001	25.45	
SLE	CRS	Simple mode	0.759	0.88078	1	0.9996	1.0003	25.45	
SLE	CRS	Weighted mode	0.857	0.89296	1	0.9997	1.0003	25.45	
CRS	SLE	MR Egger	0.411	0.69623	2E+18	8.81E-25	######	21.45	
CRS	SLE	Weighted median	0.766	0.88078	#####	3.01E-34	######	21.45	
CRS	SLE	Inverse variance weighted	0.639	0.86397	#####	2.89E-28	8E+16	21.45	
CRS	SLE	Simple mode	0.747	0.87932	1E+08	2.08E-41	######	21.45	

CRS	SLE	Weighted mode	0.873	0.89949	0.002	2.35E-36	######	21.45		
AR	CRS	MR Egger	0.138	0.37413	3069	0.11702	8E+07	50.34		
AR	CRS	Weighted median	#####	0.0004	861.1	34.2535	21647	50.34		
AR	CRS	Inverse variance weighted	#####	9.55E-10	2711	261.834	28070	50.34		
AR	CRS	Simple mode	0.134	0.37324	92.48	0.31362	27273	50.34		
AR	CRS	Weighted mode	0.062	0.2213	227.7	1.0589	48949	50.34		
CRS	AR	MR Egger	0.132	0.37324	4E+09	0.00498	######	21.44		
CRS	AR	Weighted median	0.158	0.41536	1E+06	0.00431	4E+14	21.44		
CRS	AR	Inverse variance weighted	0.287	0.57339	2369	0.00147	4E+09	21.44		
CRS	AR	Simple mode	0.812	0.89296	44.61	1.93E-12	1E+15	21.44		
CRS	AR	Weighted mode	0.128	0.37324	8E+07	0.01628	4E+17	21.44		
AT	CRS	MR Egger	#####	8.17E-08	233.4	47.3496	1150.3	77.32		
AT	CRS	Weighted median	#####	1.45E-10	39.31	14.0262	110.15	77.32		
AT	CRS	Inverse variance weighted	#####	1.81E-23	34	17.5244	65.951	77.32		
AT	CRS	Simple mode	0.015	0.0808	25.61	2.03083	323.05	77.32		
AT	CRS	Weighted mode	#####	3.00E-08	99.15	27.2249	361.1	77.32		
CRS	AT	MR Egger	0.324	0.61078	#####	1.93E-53	1E+13	21.27		
CRS	AT	Weighted median	0.636	0.86397	337.9	1.15E-08	1E+13	21.27		
CRS	AT	Inverse variance weighted	0.671	0.86397	67.43	2.41E-07	2E+10	21.27		
CRS	AT	Simple mode	0.546	0.83996	58401	3.87E-10	9E+18	21.27		
CRS	AT	Weighted mode	0.595	0.86397	12757	1.43E-10	1E+18	21.27		
PSO	CRS	MR Egger	0.134	0.37324	0.037	0.00061	2.1985	227.3		
PSO	CRS	Weighted median	0.05	0.21796	0.05	0.00248	0.9944	227.3		
PSO	CRS	Inverse variance weighted	0.05	0.21796	0.043	0.00188	1.0019	227.3		
PSO	CRS	Simple mode	0.241	0.53537	0.002	4.30E-08	52.512	227.3		
PSO	CRS	Weighted mode	0.058	0.2213	0.041	0.00195	0.8784	227.3		
CRS	PSO	MR Egger	0.296	0.57984	#####	1.40E-22	2E+06	21.34		
CRS	PSO	Weighted median	0.697	0.86397	0.008	2.12E-13	3E+08	21.34		
CRS	PSO	Inverse variance weighted	0.745	0.87932	0.064	4.17E-09	989461	21.34		
CRS	PSO	Simple mode	0.367	0.65552	#####	2.26E-24	3E+08	21.34		

CRS	PSO	Weighted mode	0.634	0.86397	0.001	2.72E-15	6E+08	21.34	
HYP	CRS	MR Egger	0.027	0.13994	12.72	1.41883	114.11	66.72	
HYP	CRS	Weighted median	0.107	0.33499	3.194	0.77753	13.118	66.72	
HYP	CRS	Inverse variance weighted	0.003	0.02088	4.849	1.71545	13.708	66.72	
HYP	CRS	Simple mode	0.666	0.86397	1.991	0.08909	44.479	66.72	
HYP	CRS	Weighted mode	0.168	0.43057	3.742	0.58613	23.896	66.72	
CRS	HYP	MR Egger	0.7	0.86397	28.56	1.53E-06	5E+08	21.34	
CRS	HYP	Weighted median	0.638	0.86397	0.051	2.16E-07	12153	21.34	
CRS	HYP	Inverse variance weighted	0.269	0.57148	0.008	1.54E-06	41.553	21.34	
CRS	HYP	Simple mode	0.696	0.86397	0.023	2.15E-10	3E+06	21.34	
CRS	HYP	Weighted mode	0.725	0.87345	0.082	8.86E-08	75079	21.34	
